# Gene–Environment Interactions in Irrational Beliefs: The Roles of Childhood Adversity and Multiple Candidate Genes

**DOI:** 10.3390/ijms25084206

**Published:** 2024-04-10

**Authors:** Adina Chiș, Lia-Ecaterina Oltean, Mirela Bîlc, Romana Vulturar, Radu Șoflău, Daniel David, Aurora Szentágotai-Tătar, Andrei C. Miu

**Affiliations:** 1 Cognitive Neuroscience Laboratory, Department of Psychology, Babeș-Bolyai University, 400015 Cluj-Napoca, Romania; adinachis82@gmail.com (A.C.); vulturar.romana@umfcluj.ro (R.V.); 2 Department of Molecular Sciences, “Iuliu Hațieganu” University of Medicine and Pharmacy, 6 Pasteur Street, 400349 Cluj-Napoca, Romania; 3 Department of Clinical Psychology and Psychotherapy, Babeș-Bolyai University, 400015 Cluj-Napoca, Romania; liaoltean@psychology.ro (L.-E.O.); radusoflau@psychology.ro (R.Ș.); danieldavid@psychology.ro (D.D.); 4 The International Institute for the Advanced Studies of Psychotherapy and Applied Mental Health, Babeș-Bolyai University, 400015 Cluj-Napoca, Romania; 5 Institute for General Practice and Interprofessional Care, University Hospital Tuebingen, 72076 Tuebingen, Germany; mire.bilc@gmail.com

**Keywords:** genetic, childhood maltreatment, maladaptive thinking, psychopathology

## Abstract

Cognitive behavioral therapy is based on the view that maladaptive thinking is the causal mechanism of mental disorders. While this view is supported by extensive evidence, very limited work has addressed the factors that contribute to the development of maladaptive thinking. The present study aimed to uncover interactions between childhood maltreatment and multiple genetic differences in irrational beliefs. Childhood maltreatment and irrational beliefs were assessed using multiple self-report instruments in a sample of healthy volunteers (*N* = 452). Eighteen single-nucleotide polymorphisms were genotyped in six candidate genes related to neurotransmitter function (*COMT*; *SLC6A4*; *OXTR*), neurotrophic factors (*BDNF*), and the hypothalamic–pituitary–adrenal axis (*NR3C1*; *CRHR1*). Gene–environment interactions (G×E) were first explored in models that employed one measure of childhood maltreatment and one measure of irrational beliefs. These effects were then followed up in models in which either the childhood maltreatment measure, the irrational belief measure, or both were substituted by parallel measures. Consistent results across models indicated that childhood maltreatment was positively associated with irrational beliefs, and these relations were significantly influenced by *COMT* rs165774 and *OXTR* rs53576. These results remain preliminary until independent replication, but they represent the best available evidence to date on G×E in a fundamental mechanism of psychopathology.

## 1. Introduction

Cognitive behavioral therapy (CBT) is the gold standard form of intervention in multiple mental disorders [1]. CBT relies on the pioneering work of Albert Ellis and Aaron Beck, who argued early after the cognitive “revolution” in psychology [2] that the causal mechanism of mental disorders is maladaptive thinking [3,4]. Ellis [4] focused on irrational beliefs, which are thought patterns that lack logical, empirical, or functional support (e.g., “I must get the approval of everybody or I am worthless”). According to this theory, irrational beliefs are activated by relevant events (e.g., social rejection) and lead to a cascade of maladaptive cognitive, behavioral, and emotional consequences [5,6]. Indeed, extensive evidence has supported the association between irrational beliefs and maladaptive cognitive processes such as dysfunctional automatic thoughts [7] and paranoid thinking [8,9]. Irrational beliefs have also been linked to maladaptive behaviors ranging from procrastination [10] to addiction and self-harm [11], as well as emotional problems such as depressive and anxiety symptoms [12]. Furthermore, clinical studies using Rational Emotive Behavior Therapy (REBT), Ellis’s pioneering form of CBT, have shown that changes in irrational beliefs are the mechanisms driving symptom remission in multiple mental disorders [13,14].

A fundamental issue that has been theoretically examined from early on in cognitive theories of psychopathology is how forms of maladaptive thinking, such as irrational beliefs, develop. Throughout his work on REBT, Ellis [4,15,16] argued that irrational beliefs have been documented across cultures and time and that their apparent universality may reflect that they are innate. He stressed that while people learn preferences and values from their parents and culture, especially in childhood, their strong tendency to overgeneralize and transform these preferences into inflexible dogmas (e.g., absolutistic “shoulds”, “oughts”, and “musts” used in interpreting themselves and the world) is largely due to innate predispositions [16]. Beck’s “generic cognitive model” proposed that maladaptive beliefs (i.e., schemas) result from the interaction of genetic factors, cognitive biases, and adverse environmental factors [17]. Genetic factors would contribute to physiological hyperactivity, which would lead to the formation of biases for emotionally relevant stimuli in attention, memory, and interpretation, which would further coalesce as schemas. Environmental factors would influence each of these processes, for instance, by shaping the valence of cognitive biases. Stressful events, in particular, would bias attention toward negatively-valenced stimuli and strengthen this bias with each repeated exposure [17]. Overall, both Ellis’s and Beck’s theories argue that maladaptive beliefs are the result of gene–environment interactions (G×E), which is in line with the dominant diathesis-stress framework for studies on psychopathology [18].

Despite the theoretical interest in the genesis of maladaptive beliefs, there is very limited empirical evidence on the role of genetic influences and stressful events (e.g., childhood maltreatment). To our knowledge, there is a single-twin study on irrational beliefs in which genetic factors were found to explain one-third of the variance, and non-shared environmental factors explained the rest of the variance [19]. On the genetic side, we are aware of a single candidate gene study, which reported an association between a polymorphism in the Catechol-O-methyltransferase (*COMT*) gene and irrational beliefs [20]. Recent evidence has also supported the association between childhood adversity and irrational beliefs [21,22], as well as two related cognitive constructs, namely maladaptive schemas [23,24,25] and maladaptive cognitions [26,27,28]. However, to our knowledge, no study until now has investigated gene–environment interactions (G×E) in irrational beliefs.

The present study investigated the relationship between childhood adversity, multiple gene polymorphisms, and irrational beliefs. We took a traditional candidate gene approach by selecting functional polymorphisms in genes coding for molecules that play a role in neural and neuroendocrine mechanisms (see Table 1). Specifically, we focused on six genes related to neurotransmitter function (i.e., *COMT*; solute carrier family 6-member 4, *SLC6A4*; oxytocin receptor, *OXTR*), neurotrophic factors (brain-derived neurotrophic factor, *BDNF*), and the hypothalamic–pituitary–adrenal axis (i.e., nuclear receptor subfamily 3 group C member 1, *NR3C1*; corticotropin-releasing hormone receptor 1, *CRHR1*). In each gene, we identified multiple single-nucleotide polymorphisms (resulting in a total of 18 polymorphisms) that could be genotyped using polymerase chain reaction methods. Overall, all the candidate genes but not all polymorphisms have been examined in relation to psychopathology (see Table 1). Some of the polymorphisms were included for methodological reasons (i.e., a polymerase chain reaction protocol was available) and helped, in our view, to account for more variance at the gene level.

**Figure 1:**
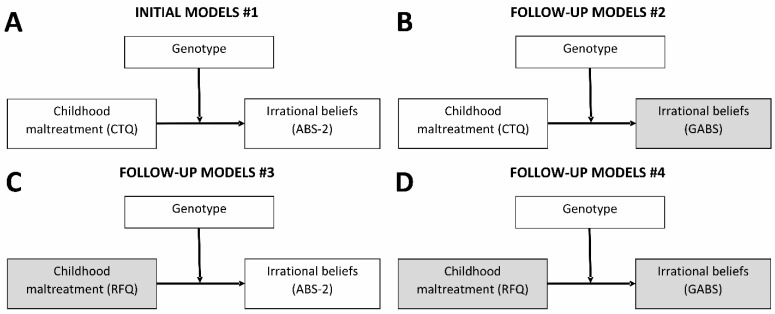
Models investigating the relations between childhood maltreatment, genotypes, and irrational beliefs: (**A**) initial models with CTQ-SF childhood maltreatment and ABS-2 irrational beliefs; (**B**) follow-up models with CTQ-SF childhood maltreatment and GABS irrational beliefs; (**C**) follow-up models with RFQ childhood maltreatment and ABS-2 irrational beliefs; and (**D**) follow-up models with RFQ childhood maltreatment and GABS irrational beliefs. Gray boxes indicate measures that were replaced relative to the initial models. Abbreviations: CTQ-SF, Childhood Trauma Questionnaire—Short Form; ABS-2, Attitude and Belief Scale-2; GABS, General Attitudes and Belief Scale; RFQ, Risky Families Questionnaire.

We employed an approach that aimed to reduce the probability of false negative findings by using two parallel measures for both childhood adversity and irrational beliefs. In the initial set of analyses, we examined the potential moderator role of genotypes in the relation between childhood adversity and irrational beliefs, using one measure from each pair. In the subsequent analyses, we sought to replicate the initial results by first using the alternative irrational belief measure, then by replacing the childhood adversity measure with the alternative scale, and finally, by replacing both the childhood adversity and the irrational belief measures (see Figure 1). We reasoned that G×E effects that remained significant in all or most of these analyses may be more reliable.

## 2. Results

### 2.1. Preliminary Analyses

#### 2.1.1. Genotype Frequencies

The genotype and allele frequencies for all polymorphisms, as well as the results of analyses on departures from the Hardy–Weinberg equilibrium, are shown in Table 2 (for further characteristics of the sample, see Supplementary Table S1). Due to missing genotypes (and some missing responses to questionnaires), the sample size varied between analyses (*N* = 301–430; see Table 2 and Supplementary Tables S3–S6). Except for *NR3C1* BclI polymorphism, all the other genotypes were in Hardy–Weinberg equilibrium. In the case of *NR3C1* N363S and *NR3C1* ER22/23EK polymorphisms, there were no homozygotes for the minor allele. Similarly, for *OXTR* rs2254298 and *CRHR1* rs242938, the number of homozygotes for the minor allele was very low (i.e., <1% of the sample). Considering that these genotypes were in the Hardy–Weinberg equilibrium, they were included in the subsequent analyses but limited to two genotypes (i.e., major allele homozygotes and heterozygotes).

#### 2.1.2. Correlational Analysis

As expected, the scores on the two childhood adversity measures (i.e., CTQ-SF and RFQ) showed high correlations: *r*[444] = 0.80, *p* < 0.001. Similarly high correlations were found between the two irrational belief measures (i.e., ABS-2 and GABS): *r*[445] = 0.87, *p* < 0.001. These correlations supported our hypothesis that these measures largely overlapped and could be treated as parallel measures of the same construct.

Table 3 shows the correlations between childhood adversity, irrational beliefs, and depressive and generalized anxiety symptoms. As expected, there were significant positive correlations between both childhood adversity measures and depressive and generalized anxiety symptoms, as well as both irrational belief measures and depressive and generalized anxiety symptoms (for correlations between childhood adversity and specific irrational beliefs, see Supplementary Table S2).

We also examined potential associations between genotypes and childhood adversity. With one exception, all correlations between the genotypes and both CTQ-SF (all *p*s ≥ 0.293) and RFQ (all *p*s ≥ 0.115) were not significant. The exception was *NR3C1* ER22/23EK, in which case participants with the GA genotype reported higher CTQ-SF scores compared with those with the GG genotype.

### 2.2. Gene–Environment Interactions

**Figure 2:**
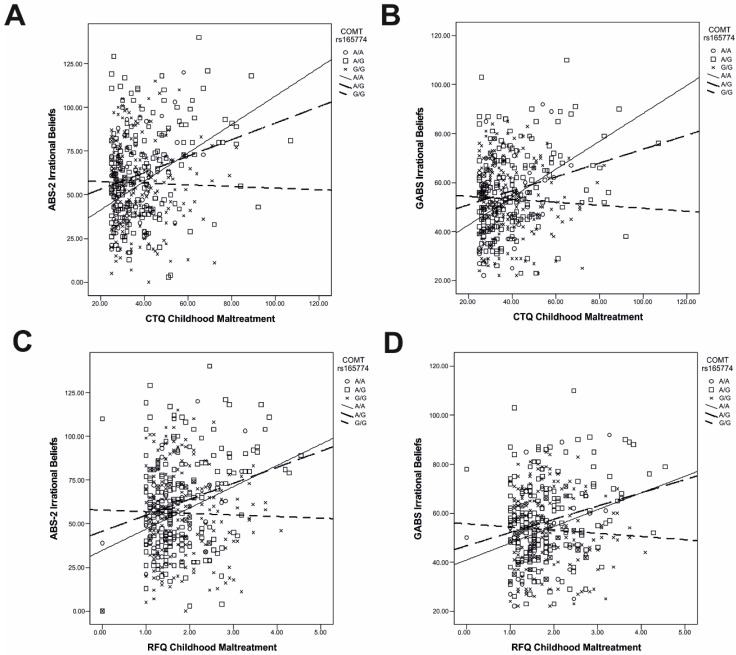
The moderator role of *COMT* rs165774 in the relationship between childhood maltreatment and irrational beliefs, as reflected by different measures: (**A**) CTQ-SF childhood maltreatment and ABS-2 irrational beliefs; (**B**) CTQ-SF childhood maltreatment and GABS irrational beliefs; (**C**) RFQ childhood maltreatment and ABS-2 irrational beliefs; and (**D**) RFQ childhood maltreatment and GABS irrational beliefs. Abbreviations: CTQ-SF, Childhood Trauma Questionnaire—Short Form; ABS-2, Attitude and Belief Scale-2; GABS, General Attitudes and Belief Scale; RFQ, Risky Families Questionnaire.

The first set of models focused on the moderator role of each genotype in the relationship between CTQ-SF childhood maltreatment and ABS-2 irrational beliefs (see Supplementary Table S3). In all models but two, childhood maltreatment was positively associated with irrational beliefs (all significant: *p*s ≤ 0.050) (see Supplementary Table S3). Furthermore, one of the interactions between *COMT* rs165774 (dummy 2: GG vs. AA and AG) and childhood maltreatment was significant for irrational beliefs (*B* = −0.86; SE(*B*) = 0.38; *p* = 0.025; 95% CI: −1.61, −0.10) (see Supplementary Table S3). Slope analysis indicated that the relationship between childhood maltreatment and irrational beliefs was significant in the AA (*p* = 0.021) and AG (*p* < 0.001) genotypes but not in the GG genotype (*p* = 0.757) (see Figure 2A).

**Figure 3:**
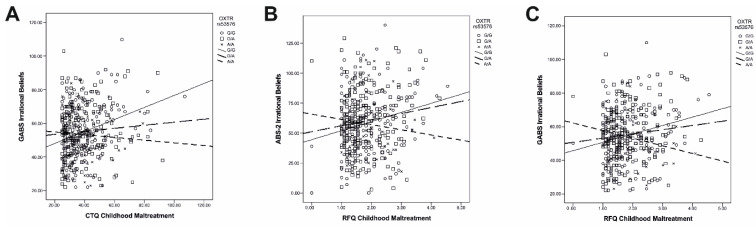
The moderator role of *OXTR* rs53576 in the relationship between childhood maltreatment and irrational beliefs, as reflected by different measures: (**A**) CTQ-SF childhood maltreatment and GABS irrational beliefs; (**B**) RFQ childhood maltreatment and ABS-2 irrational beliefs; and (**C**) RFQ childhood maltreatment and GABS irrational beliefs. Abbreviations: CTQ-SF, Childhood Trauma Questionnaire—Short Form; ABS-2, Attitude and Belief Scale-2; GABS, General Attitudes and Belief Scale; RFQ, Risky Families Questionnaire.

The second set of models examined the moderator role of each genotype in the relationship between CTQ-SF childhood maltreatment and GABS irrational beliefs (see Supplementary Table S4). In other words, we sought to conceptually replicate the results in the first set of models by using the parallel measure of irrational beliefs as the criterion variable. We replicated the positive association between childhood maltreatment and irrational beliefs in all but one model (all significant: *p*s ≤ 0.049) (see Supplementary Table S4). *COMT* rs165774 × childhood maltreatment was also significant (*B* = −0.63; SE(*B*) = 0.23; *p* = 0.008; 95% CI: −1.09, −0.16) (see Supplementary Table S4), with the positive association between childhood maltreatment and irrational beliefs being significant in the AA (*p* = 0.009) and AG (*p* < 0.001) genotypes but not the GG genotype (*p* = 0.518) (Figure 2B). In addition, the interactions between both the dummy variables contrasting *OXTR* rs53576 genotypes and childhood maltreatment were significant: dummy 1 (AG vs. GG & AA): *B* = −0.26; SE(*B*) = 0.12; *p* = 0.026; 95% CI: −0.50, −0.03; dummy 2 (AA vs. GG & AG): *B* = −0.43; SE(*B*) = 0.19; *p* = 0.023; 95% CI: −0.81, −0.06 (see Supplementary Table S4). Slope analysis indicated that the positive association between childhood maltreatment and irrational beliefs was significant in the GG (*p* < 0.001) genotype but not in the AG (*p* = 0.276) and AA (*p* = 0.636) genotypes (Figure 3A).

In the third set of models, we investigated the moderator role of genotypes in the relationship between RFQ childhood maltreatment and ABS-2 irrational beliefs. These models represented another attempt to replicate the results in the original models by using the parallel measure of childhood maltreatment as the predictor variable. In all but five models, there was a significant positive association between childhood maltreatment and irrational beliefs (all significant: *p*s ≤ 0.046) (see Supplementary Table S5). The interaction between *COMT* rs165774 and childhood maltreatment was again significant (*B* = −13.06; SE(*B*) = 6.38; *p* = 0.042; 95% CI: −25.62, −0.50) (see Supplementary Table S5), with the positive relationship between childhood maltreatment and irrational beliefs being significant in the AA (*p* = 0.038) and AG (*p* < 0.001) genotypes but not in the GG genotype (*p* = 0.722) (Figure 2C). Furthermore, *OXTR* rs53576 × childhood maltreatment was also significant (*B* = −11.92; SE(*B*) = 5.61; *p* = 0.034; 95% CI: −22.96, −0.89) (see Supplementary Table S5): the positive association between childhood maltreatment and irrational beliefs was significant in the GG genotype (*p* = 0.002) but not in the AG (*p* = 0.057) and AA (*p* = 0.387) genotypes (Figure 3B). A new interaction that was significant was *BDNF* rs6265 × childhood maltreatment (*B* = 19.74; SE(*B*) = 8.90; *p* = 0.027; 95% CI: 2.21, 37.28) (see Supplementary Table S5): the positive association between childhood maltreatment and irrational beliefs was significant in the GG (*p* = 0.015) and AA (*p* = 0.002) genotypes but not the GA genotype (*p* = 0.973).

The final set of follow-up models focused on the moderator role of genotypes in the relationship between RFQ childhood maltreatment and GABS irrational beliefs. In these models, we sought to replicate the original results by replacing both the predictor and the criterion variables with parallel measures. The positive association between childhood maltreatment and irrational beliefs was significant in all but seven models (all significant: *p*s ≤ 0.029) (see Supplementary Table S6). The interaction between *COMT* rs165774 and childhood maltreatment was significant (*B* = −8.14; SE(*B*) = 3.95; *p* = 0.040; 95% CI: −15.91, −0.37) (see Supplementary Table S6). The slope analysis showed that the positive association between childhood maltreatment and irrational beliefs was significant in the AG genotype (*p* < 0.001) but not in the GG genotype (*p* = 0.426); the association just fell short of significance in the AA genotype (*p* = 0.058) (Figure 2D). *OXTR* rs53576 × childhood maltreatment was significant (*B* = −9.54; SE(*B*) = 3.47; *p* = 0.006; 95% CI: −16.37, −2.70) (see Supplementary Table S6), with the association between childhood maltreatment and irrational beliefs being significant in the GG (*p* = 0.002) but not the AG (*p* = 0.123) and AA (*p* = 0.142) genotypes (Figure 3C). The *BDNF* rs6265 × childhood maltreatment approached but fell short of significance (*p* = 0.085) (see Supplementary Table S6).

The small number of men in the sample did not allow for investigating G×E×sex effects. However, in order to control for potential sex differences, we followed up on all significant G×E effects in the larger subsample of women. All the effects remained significant.

## 3. Discussion

The results of the present study indicate that a history of childhood maltreatment is associated with higher levels of irrational beliefs in adulthood and that this relationship may be influenced by certain genotypes. Specifically, the association between childhood maltreatment and irrational beliefs was apparent in the AA and AG genotypes but not in the GG genotype of *COMT* rs165774. Similarly, childhood maltreatment was associated with irrational beliefs in the GG genotype but not in the AG and AA genotypes of *OXTR* rs53576. These G×E effects were conceptually replicated in multiple models in which measures of either childhood maltreatment, irrational beliefs, or both were substituted by parallel measures.

The present results support the theoretical view that childhood adversity is related to maladaptive thinking [16,17]. We found a positive association between a history of childhood maltreatment and irrational beliefs, which is in line with previous studies [21,22]. The magnitude of this association was small, both at the levels of global irrationality (i.e., *r*s between 0.12 and 0.15; see Table 3) and specific irrational beliefs (i.e., *r*s between 0.04 and 0.21; see Supplementary Table S2). One explanation for the small effect size is the focus on a healthy sample of students, showing limited variance in symptoms of psychopathology. Another explanation that is in line with the G×E view proposed in cognitive theories of psychopathology [17] underscores the involvement of moderator variables, such as genetic differences.

From a candidate gene perspective, the moderator role of *COMT* and *OXTR* gene polymorphisms could be justified based on the role of the corresponding molecules in brain processes. COMT is an enzyme that catalyzes catechol-containing substrates, such as the catecholamine neurotransmitters [37]. Knockout mice for the *COMT* gene showed a 60% increase in extracellular dopamine levels in the prefrontal cortex compared with wild-type mice [38]. In addition to higher levels of anxiety, they showed differential physiological responses to acute and chronic stress (e.g., [39]). *OXTR* encodes the receptor for oxytocin, a hormone and neurotransmitter produced in the hypothalamus, and is expressed widely in the human brain [40]. Knockout mice for the *OXTR* gene are characterized by deficits in social recognition and higher levels of aggressive behavior [41,42]. Overall, this evidence from animal models suggests that low levels of COMT and OXTR are associated with behavioral problems. For *COMT* rs165774, the A allele is associated with lower COMT activity compared with the G allele [31]. In line with this view, the present results suggest that A-allele carriers may be more vulnerable to childhood adversity, as indicated by their higher levels of irrational beliefs (but see [43]). The polymorphism *OXTR* rs5357 has not been functionally characterized, but a previous candidate gene study found that childhood maltreatment was associated with depressive symptoms only in G-allele carriers [44]. In line with this view, the present study found that childhood maltreatment may be associated with irrational beliefs only in G-allele homozygotes.

In our view, one of the strong points of this study is the conceptual replication of initial results by employing parallel measures of both childhood maltreatment and irrational beliefs. While this may have indeed allowed us to uncover real G×E effects, it is also possible that it reflects intrinsic limitations (e.g., social desirability) common to all self-report measures of childhood maltreatment and irrational beliefs, or it reflects characteristics of this convenience sample, which is not representative of the population. Furthermore, we have to ponder whether the candidate gene approach is able to uncover G×E effects in a sample of this size. On the encouraging side, the Monte Carlo simulations presented by Duncan and Keller [45] suggested that a large effect size, such as that of *COMT* rs165774 × childhood maltreatment in the present study, which explained between 2.1 and 2.6% in all the models, would require a sample such as the one in this study. On a less encouraging side, recent genome-wide association studies (GWASs) have suggested that samples need to be much larger, of at least tens of thousands of participants, in order to have sufficient power to detect any G×E effect [46]. In the same vein, not correcting the statistical analyses for multiple testing is another limitation. Mindful of the low replicability of candidate gene approaches in relatively small samples [46,47,48], we put forward the present results as a preliminary. However, considering that GWASs have not yet approached mechanisms of psychopathology, such as maladaptive beliefs, the present results stand as the best available evidence to date for G×E in this domain. From a clinical point of view, the present results suggest that individual differences in childhood maltreatment and genetic differences could be considered predictors of the response to interventions targeting maladaptive beliefs, such as CBT.

## 4. Material and Methods

### 4.1. Participants

Four hundred and fifty-two volunteers (84.73% women; age 19.58 ± 3.53 years) participated in this study. All participants were Caucasians of European descent, and Romanian was their first language. They were recruited through campus and online advertisements in three waves (2016, 2017, and 2018), and they were all students at Babeș-Bolyai University. Self-reported parental education was as follows: less than high school (7.8%), high school (42.9%), and Bachelor’s studies (49.3%). None of the participants reported neuropsychiatric disorders or using psychoactive medication.

### 4.2. Measures

#### 4.2.1. Genotyping

DNA was extracted from buccal epithelial cells using the MasterPureTM Complete DNA & RNA Purification Kit (Epicentre, Madison, WI, USA) and kept at −20 °C. Seventeen genetic polymorphisms in six genes were genotyped (see Supplementary Images): (1) *COMT* gene polymorphisms rs6269, rs737865, rs165774, rs2075507, and rs4818; (2) *NR3C1* gene polymorphisms BclI, N363S, and ER22/23EK; (3) *OXTR* gene polymorphisms rs2254298 and rs53576; (4) *CRHR1* gene polymorphism rs242938; (5) *SLC6A4* gene polymorphisms *5-HTTLPR* and rs25531; and (6) *BDNF* gene polymorphisms rs6265, rs988748, rs7103411, rs1103014, and rs11757. The genotyping method was polymerase chain reaction-restriction fragment length polymorphism (PCR-RFLP) for the *NR3C1* [34], *OXTR* [49], *CRHR1* [50], and *SLC6A4* [51] polymorphisms and tetra-primer amplification refractory mutation system-polymerase chain reaction (T-ARMS-PCR) for the *COMT* [52] and *BDNF* [53] polymorphisms. In the present sample, the allelic frequencies were comparable to those reported in the dbSNP database (see Supplementary Table S7).

#### 4.2.2. Childhood Adversity

Childhood adversity was assessed using two questionnaires: Childhood Trauma Questionnaire—Short Form (CTQ-SF) [54] and Risky Families Questionnaire (RFQ) [55].

CTQ-SF is a 25-item self-report measure that examines exposure to five types of childhood maltreatment (i.e., emotional, physical, and sexual abuse and physical and emotional neglect), rated on a five-point Likert scale (0 = *never true* to 4 = *very often*). In this study, we focused on the total maltreatment score, which showed very good reliability in this sample (Cronbach’s alpha = 0.91).

RFQ is an 11-item self-report questionnaire that examines exposure to four forms of childhood adversity (i.e., abuse, neglect, family conflict, and household disorganization), rated on a four-point Likert scale (1 = *rarely or none of the time* to 4 = *most or all of the time*). This study focused on the total score, which showed good reliability in this sample (Cronbach’s alpha = 0.79).

#### 4.2.3. Irrational Beliefs

Irrational beliefs were assessed using the Attitude and Belief Scale-2 (ABS-2) [56] and the General Attitudes and Belief Scale (GABS) [57].

ABS-2 is a 72-item self-reported scale that assesses three factors: (1) irrational and rational beliefs; (2) cognitive processes (i.e., demandingness, awfulizing, frustration intolerance, and self-downing); and (3) content/context information (i.e., beliefs about achievement, affiliation, and comfort). Each item is rated on a five-point Likert scale (A = *strong disagreement* to E = *strong agreement*). For the present study, we focused on the total irrationality score, which showed very good reliability in this sample (Cronbach’s alpha = 0.96).

GABS is a 26-item self-report questionnaire that assessed both irrational beliefs (i.e., demandingness, awfulizing, low frustration tolerance, and self-downing) and rational beliefs (i.e., needs for achievement, approval, and comfort and demands for fairness). In the present study, we focused on the total irrationality score, which showed good reliability in this sample (Cronbach’s alpha = 0.88).

### 4.3. Statistical Analysis

We employed the typical analysis employed in this kind of G×E studies, with continuous variables for the predictor (i.e., childhood adversity) and the outcome (i.e., irrational beliefs) and a categorical variable for the moderator (i.e., genotype) (for other illustrations of the design and analysis see [58,59,60,61]).

First, we examined departures from the Hardy–Weinberg equilibrium for all genotypes to determine whether genotype frequencies in the present sample were significantly different from the population [62]. Only genotypes not significantly departing from the Hardy–Weinberg equilibrium were subsequently analyzed.

In the next step, we examined the correlations between the two parallel measures of childhood adversity questionnaires, on the one hand, and irrational beliefs, on the other hand. Since each of these pairs of scales assesses the same construct, we expected high correlations (i.e., *r* ≥ 0.7) between them. Provided that those correlations supported a high degree of overlap, we planned to use one of the scales in each pair for exploratory analyses and the other for conceptual replication.

Another preliminary analysis involved examining potential gene–environment correlations (i.e., genotypes and childhood adversity) that can be a confound in G×E analyses [63].

The preliminary analyses also examined the associations between irrational beliefs and depressive and generalized anxiety symptoms, as well as the associations between childhood adversity and depressive and generalized anxiety symptoms.

The main analyses focused on examining the moderator role of each genotype in the relationship between childhood adversity and irrational beliefs using multiple regression analyses. The first modes focused on (1) investigating CTQ-SF × genotype on ABS-2 irrational beliefs and (2) conceptually replicating the effect of CTQ-SF × genotype on GABS, the parallel measure of irrational beliefs (see Figure 1). These analyses were followed up by similar analyses in which RFQ was the childhood adversity measure, that is, (3) investigating RFQ × genotype on ABS-2 irrational beliefs and (4) conceptually replicating the effect of RFQ × genotype on GABS, the parallel measure of irrational beliefs (see Figure 1).

All the genotypes meeting the criterion for analysis (i.e., not significantly departing from the Hardy–Weinberg equilibrium) were dummy-coded, taking the larger group of homozygotes as the comparison group. Where there were three genotypes, we created two dummy variables contrasting the homozygotes for the minor allele and the heterozygotes, respectively, with the comparison group (see Supplementary Tables S2–S5). Continuous predictors (i.e., CTQ-SF and RFQ) were centered before being entered into the regression model. Significant interactions were followed up by slope analysis, examining the relation between the predictor and the criterion variables in each moderator category.

All analyses were run in SPSS (IBM, Armonk, NY, USA), including the PROCESS 4.0 macro for SPSS [64].

## 5. Conclusions

The present results suggest that two polymorphisms, *COMT* rs165774 and *OXTR* rs5357, may play a moderator role in the relation between childhood maltreatment and irrational beliefs. This study represents the first attempt to uncover G×E in irrational beliefs, and we hope that it will stimulate further work, ideally at the genome-wide level, on this fundamental mechanism of psychopathology.

## Figures and Tables

**Table 1:** Description of the genetic polymorphisms genotyped in the present study (see also Supplementary Images and Supplementary Table S7).

Gene	Position on Chromosome, RefSeq	RefSNPID	Ancestral Allele (A1)	Minor Allele (A2)	Allele Functional Characterization	References
*SLC6A4*	chr17, NC_000017.11:g.30237328T>C (promoter region)	rs25531 (*5-HTTLPR*)	T (or L)	C (or S)	The short (C or S) allele is associated with reduced serotonin transporter expression and function compared with the long allele (T or L).	[29]
*COMT*	chr22, NC_000022.11:g.19962429A>G (promoter region)	rs6269	A	G	The G allele is associated with lower expression of *COMT* mRNA in the human brain.	[30]
	chr22, NC_000022.11:g.19942598A>G, (intron region)	rs737865	A	G	The functionality is unknown.	-
	chr22, NC_000022.11:g.19965038G>A (3′-untranslated region)	rs165774	G	A	The minor A allele is associated with a increased mRNA stability and with substantially decreased activity of the product molecule, most likely through modulation of translational efficiency.	[31]
	chr22, NC_000022.11:g.19940569G>A (promoter region)	rs2075507	G	A	The G allele may be associated with reduced *COMT* activity.	[32]
	chr22, NC_000022.11:g.19963684C>G (coding region)	rs4818	C	G	It is a synonymous polymorphism, but evidence suggests that the C allele is associated with a lower enzyme activity.	[33]
*BDNF*	chr11, NC_000011.10:g.27658369C>T (coding region)	rs6265	G	A	The A allele is associated with a lower expression of BDNF mRNA.	[32]
	chr11, NC_000011.10:g.27703198C>G (intron region)	rs988748	C	G	The functionality is unknown.	-
	chr11, NC_000011.10:g.27678578C>T (intron region)	rs7103411	C	T	The functionality is unknown.	-
	chr11, NC_000011.10:g.27662970A>G (intron region)	rs11030104	T	C	The functionality is unknown.	-
	chr11, NC_000011.10:g.27646808G>C (intron region)	rs16917204 (rs11757)	G	C	The functionality is unknown.	-
*CRHR1*	chr17, NC_000017.11:g.45818570A>C (intron region)	rs242938	A	G	The functionality is unknown.	-
*NR3C1*	chr5, NC_000005.10:g.143399010G>C (intron 2)	rs41423247 (or BclI)	C	G	The sensitivity to glucocorticoids appears to be different and tissue-specific.	[34]
	chr5, NC_000005.10:g.143400774C>A/ NC_000005.10:g.143400772C>G (coding region)	rs6189/rs6190 (ER22/23EK)	G	A	The first variant is silent. The second variant results in arginine (R) to lysine (K) change. The presence of the ER22/23EK A allele is related to resistance to glucocorticoids.	
	chr5, NC_000005.10:g.143399752T>A (coding region)	rs56149945 (N363S)	A	G	The G allele is associated with increased sensitivity to glucocorticoids.	
*OXTR*	chr3, NC_000003.12:g.8762685A>G (intron region)	rs53576	A	G	The functionality is unknown.	[35]
	chr3, NC_000003.12:g.8760542G>A (intron region)	rs2254298	G	A	The functionality is unknown.	[36]

Note: A, adenine; *BDNF*, brain-derived neurotrophic factor gene; C, cytosine; chr, chromosome; *COMT*, Catechol-O-methyltransferase gene; *CRHR1*, corticotropin-releasing hormone receptor 1 gene; G, guanine; L, long allele; NC, reference sequence, complete genomic molecule; *NR3C1*, nuclear receptor subfamily 3 group C member 1 gene; *OXTR*, oxytocin receptor gene; rs, reference SNP cluster ID; S, short allele; *SLC6A4*, solute carrier family 6-member 4 gene; T, thymine.

**Table 2:** Genotype and allele distribution and the Hardy–Weinberg equilibrium for polymorphisms genotyped in the present study.

Gene Polymorphism (*N*)	Genotype (Frequency %)			Allele (Frequency %)		Hardy–Weinberg Equilibrium
*COMT* rs6269 (*N* = 309)	AA (37.54)	AG (44.66)	GG (17.8)	A (59.87)	G (40.13)	*χ*^2^ = 1.53, *p* = 0.214
*COMT* rs737865 (*N* = 438)	AA (47.72)	AG (42.01)	GG (10.27)	A (68.73)	G (31.27)	*χ*^2^ = 0.22, *p* = 0.633
*COMT* rs165774 (*N* = 438)	AA (9.36)	AG (44.52)	GG (46.12)	A (31.62)	G (68.38)	*χ*^2^ = 0.38, *p* = 0.536
*COMT* rs2075507 (*N* = 309)	AA (18.77)	AG (43.69)	GG (37.54)	A (40.62)	G (59.38)	*χ*^2^ = 2.74, *p* = 0.097
*COMT* rs4818 (*N* = 309)	CC (39.48)	CG (42.72)	GG (17.8)	C (60.84)	G (39.16)	*χ*^2^ = 3.30, *p* = 0.068
*NR3C1* BclI (*N* = 309)	CC (46.6)	CG (38.83)	GG (14.56)	C (66.10)	G (33.90)	*χ*^2^ = 5.58, *p* = 0.018
*NR3C1* N363S (*N* = 308)	AA (94.16)	AG (5.84)	GG (0)	A (97.08)	G (2.92)	*χ*^2^ = 0.27, *p* = 0.597
*NR3C1* ER22/23EK (*N* = 309)	GG (95.15)	GA (4.85)	AA (0)	G (97.58)	A (2.42)	*χ*^2^ = 0.19, *p* = 0.661
*OXTR* rs2254298 (*N* = 309)	GG (85.52)	AG (16.5)	AA (0.97)	G (93.77)	A (9.23)	*χ*^2^ = 0.06, *p* = 0.661
*OXTR* rs53576 (*N* = 438)	GG (45.89)	AG (43.15)	AA (10.96)	G (67.47)	A (32.54)	*χ*^2^ = 0.12, *p* = 0.721
*CRHR1* rs242938 (*N* = 309)	GG (84.14)	GA (15.53)	AA (0.32)	G (91.91)	A (8.09)	*χ*^2^ = 0.61, *p* = 0.434
*SLC6A4 5HTTLPR*/rs25531 (*N* = 309)	L′/L′ (24.27)	L′/S′ (51.46)	S′/S′ (24.27)	L′ (50)	S′ (50)	*χ*^2^ = 0.09, *p* = 0.763
*BDNF* rs6265 (*N* = 309)	GG (60.84)	GA (33.66)	AA (5.50)	G (77.67)	A (22.33)	*χ*^2^ = 0.27, *p* = 0.763
*BDNF* rs988748 (*N* = 309)	GG (55.99)	CG (37.22)	CC (6.80)	G (74.60)	C (25.40)	*χ*^2^ = 0.10, *p* = 0.750
*BDNF* rs7103411 (*N* = 309)	TT (58.90)	TC (37.54)	CC (3.56)	T (77.67)	C (22.33)	*χ*^2^ = 2.09, *p* = 0.148
*BDNF* rs1103014 (*N* = 309)	TT (55.99)	TC (35.92)	CC (8.09)	T (73.95)	C (26.05)	*χ*^2^ = 1.41, *p* = 0.234
*BDNF* rs11757 (*N* = 309)	GG (55.02)	CG (37.54)	CC (7.44)	G (73.79)	C (26.21)	*χ*^2^ = 0.27, *p* = 0.603

Note: A, adenine; *BDNF*, brain-derived neurotrophic factor gene; C, cytosine; chr, chromosome; *COMT*, Catechol-O-methyltransferase gene; *CRHR1*, corticotropin-releasing hormone receptor 1 gene; G, guanine; L, long allele; NC, reference sequence, complete genomic molecule; *NR3C1*, nuclear receptor subfamily 3 group C member 1 gene; *OXTR*, oxytocin receptor gene; rs, reference SNP cluster ID; S, short allele; *SLC6A4*, solute carrier family 6-member 4 gene; T, thymine.

**Table 3:** Correlations between childhood adversity, irrational beliefs, and depressive and generalized anxiety symptoms.

	CTQ-SF Total Score	RFQ Total Score	ABS-2 Total Irrationality Score	GABS Total Irrationality Score	PHQ-9 Score	GAD-7 Score
CTQ-SF total score	-	0.80 **	0.15 **	0.15 **	0.35 **	039 **
RFQ total score		-	0.13 **	0.12 **	0.23 **	0.26 **
ABS-2 irrationality score			-	0.87 **	0.42 **	0.47 **
GABS irrationality score				-	0.38 **	0.47 **
PHQ-9					-	0.80

Note: CTQ-SF, Childhood Trauma Questionnaire—Short Form; ABS-2, Attitude and Belief Scale-2; GABS, General Attitudes and Belief Scale; RFQ, Risky Families Questionnaire. ** *p* < 0.01.

## Data Availability

Data are contained within the article and the Supplementary Materials.

## Supplementary Materials

The supporting information can be downloaded at https://www.mdpi.com/article/10.3390/ijms25084206/s1.

## References

[1] David D., Cristea I., Hofmann S.G. (2018). Why cognitive behavioral therapy is the current gold standard of psychotherapy. Front. Psychiatry.

[2] Miller G.A. (2003). The cognitive revolution: A historical perspective. Trends Cogn. Sci..

[3] Beck A.T. (1967). Depression. Clinical, Experimental and Theoretical Aspects.

[4] Ellis A. (1962). Reason and Emotion in Psychotherapy.

[5] David D., Cardoș R., Cândea D., Oltean H., Ştefan S., Dryden W., Bernard M.E. (2019). REBT and Depressive Disorders. REBT with Diverse Client Problems and Populations.

[6] Ellis A. (1995). Thinking processes involved in irrational beliefs and their disturbed consequences. J. Cogn. Psychother..

[7] Soflau R., David D. (2017). A meta-analytical approach of the relationships between the irrationality of beliefs and the functionality of automatic thoughts. Cogn. Ther. Res..

[8] Soflau R., David D. (2019). The impact of irrational beliefs on paranoid thoughts. Behav. Cogn. Psychother..

[9] Vukosavljevic-Gvozden T., Filipovic S., Opacic G. (2015). The mediating role of symptoms of psychopathology between irrational beliefs and Internet gaming addiction. J. Ration.-Emotive Cogn.-Behav. Ther..

[10] Balkis M., Duru E. (2019). Procrastination and rational/irrational beliefs: A moderated mediation model. J. Ration.-Emotive Cogn.-Behav. Ther..

[11] Szentagotai-Tatar A., Freeman A. (2007). An analysis of the relationship between irrational beliefs and automatic thoughts in predicting distress. J. Cogn. Behav. Psychother..

[12] Vîslă A., Flückiger C., Grosse Holtforth M., David D. (2016). Irrational beliefs and psychological distress: A meta-analysis. Psychother. Psychosom..

[13] David D., Lynn S.J., Ellis A. (2009). Rational and Irrational Beliefs: Research, Theory, and Clinical Practice.

[14] David D., Szentagotai A., Lupu V., Cosman D. (2008). Rational emotive behavior therapy, cognitive therapy, and medication in the treatment of major depressive disorder: A randomized clinical trial, posttreatment outcomes, and six-month follow-up. J. Clin. Psychol..

[15] Ellis A. (1976). The biological basis of human irrationality. J. Individ. Psychol. Health.

[16] Ellis A. (1990). Is rational-emotive therapy (RET)“rationalist” or “constructivist”?. J. Ration.-Emotive Cogn.-Behav. Ther..

[17] Beck A.T., Haigh E.A. (2014). Advances in cognitive theory and therapy: The generic cognitive model. Annu. Rev. Clin. Psychol..

[18] Monroe S.M., Simons A.D. (1991). Diathesis-stress theories in the context of life stress research: Implications for the depressive disorders. Psychol. Bull..

[19] Chen J., Li X. (2014). Genetic and environmental etiologies of adolescent dysfunctional attitudes: A twin study. Twin Res. Hum. Genet..

[20] Podina I., Popp R., Pop I., David D. (2015). Genetic correlates of maladaptive beliefs: COMT Val158Met and irrational cognitions linked depending on distress. Behav. Ther..

[21] Duru E., Balkis M. (2022). Childhood trauma, depressive symptoms and rational/irrational beliefs: A moderated mediation model. Curr. Psychol..

[22] Soflau R., Szentagotai-Tatar A., Oltean L.E. (2023). Childhood adversity, resilience, and paranoia during the COVID-19 outbreak. The mediating role of irrational beliefs and affective disturbance. J. Ration.-Emotive Cogn.-Behav. Ther..

[23] Estévez A., Ozerinjauregi N., Herrero-Fernández D., Jauregui P. (2016). The mediator role of early maladaptive schemas between childhood sexual abuse and impulsive symptoms in female survivors of CSA. J. Interpers. Violence.

[24] Feyzioğlu A., Taşlıoğlu Sayıner A.C., Özçelik D., Tarımtay Altun F., Budak E.N. (2023). The mediating role of early maladaptive schemas in the relationship between early childhood trauma and alexithymia. Curr. Psychol..

[25] Wright M.O.D., Crawford E., Del Castillo D. (2009). Childhood emotional maltreatment and later psychological distress among college students: The mediating role of maladaptive schemas. Child Abus. Negl..

[26] Berman I.S., Petretric P., Bridges A.J. (2021). Beyond child maltreatment: The incremental value of household dysfunction in the prediction of negative beliefs and internalizing symptoms in women. J. Am. Coll. Health.

[27] Gibb B.E., Alloy L.B., Abramson L.Y., Rose D.T., Whitehouse W.G., Donovan P., Hogan M.E., Cronholm J., Tierney S. (2001). History of childhood maltreatment, negative cognitive styles, and episodes of depression in adulthood. Cogn. Ther. Res..

[28] Kaysen D., Scher C.D., Mastnak J., Resick P. (2005). Cognitive mediation of childhood maltreatment and adult depression in recent crime victims. Behav. Ther..

[29] Lesch K.P., Bengel D., Heils A., Sabol S.Z., Greenberg B.D., Petri S., Benjamin J., Muller C.R., Hamer D.H., Murphy D.L. (1996). Association of anxiety-related traits with a polymorphism in the serotonin transporter gene regulatory region. Science.

[30] Gaysina D., Xu M.K., Barnett J.H., Croudace T.J., Wong A., Richards M., Jones P.B., The LHA Genetics Group (2013). The catechol-O-methyltransferase gene (COMT) and cognitive function from childhood through adolescence. Biol. Psychol..

[31] Meloto C.B., Segall S.K., Smith S., Parisien M., Shabalina S.A., Rizzatti-Barbosa C.M., Gauthier J., Tsao D., Convertino M., Piltonen M.H. (2015). COMT gene locus: New functional variants. Pain.

[32] Chen J., Lipska B.K., Halim N., Ma Q.D., Matsumoto M., Melhem S., Kolachana B.S., Hyde T.M., Herman M.M., Apud J. (2004). Functional analysis of genetic variation in catechol-O-methyltransferase (COMT): Effects on mRNA, protein, and enzyme activity in postmortem human brain. Am. J. Hum. Genet..

[33] Roussos P., Giakoumaki S.G., Pavlakis S., Bitsios P. (2008). Planning, decision-making and the COMT rs4818 polymorphism in healthy males. Neuropsychologia.

[34] Galecka E., Szemraj J., Bienkiewicz M., Majsterek I., Przybylowska-Sygut K., Galecki P., Lewinski A. (2013). Single nucleotide polymorphisms of NR3C1 gene and recurrent depressive disorder in population of Poland. Mol. Biol. Rep..

[35] Tops S., Habel U., Radke S. (2019). Genetic and epigenetic regulatory mechanisms of the oxytocin receptor gene (OXTR) and the (clinical) implications for social behavior. Horm. Behav..

[36] Camerini L., Zurchimitten G., Bock B., Xavier J., Bastos C.R., Martins E., Ardais A.P., Dos Santos Motta J.V., Pires A.J., de Matos M.B. (2022). Genetic variations in elements of the oxytocinergic pathway are associated with attention/hyperactivity problems and anxiety problems in childhood. Child Psychiatry Hum. Dev..

[37] Mannisto P.T., Kaakkola S. (1999). Catechol-O-methyltransferase (COMT): Biochemistry, molecular biology, pharmacology, and clinical efficacy of the new selective COMT inhibitors. Pharmacol. Rev..

[38] Kaenmaki M., Tammimaki A., Myohanen T., Pakarinen K., Amberg C., Karayiorgou M., Gogos J.A., Mannisto P.T. (2010). Quantitative role of COMT in dopamine clearance in the prefrontal cortex of freely moving mice. J. Neurochem..

[39] Desbonnet L., Tighe O., Karayiorgou M., Gogos J.A., Waddington J.L., O’Tuathaigh C.M. (2012). Physiological and behavioural responsivity to stress and anxiogenic stimuli in COMT-deficient mice. Behav. Brain Res..

[40] Quintana D.S., Rokicki J., van der Meer D., Alnaes D., Kaufmann T., Cordova-Palomera A., Dieset I., Andreassen O.A., Westlye L.T. (2019). Oxytocin pathway gene networks in the human brain. Nat. Commun..

[41] Lee H.J., Caldwell H.K., Macbeth A.H., Tolu S.G., Young W.S. (2008). A conditional knockout mouse line of the oxytocin receptor. Endocrinology.

[42] Takayanagi Y., Yoshida M., Bielsky I.F., Ross H.E., Kawamata M., Onaka T., Yanagisawa T., Kimura T., Matzuk M.M., Young L.J. (2005). Pervasive social deficits, but normal parturition, in oxytocin receptor-deficient mice. Proc. Natl. Acad. Sci. USA.

[43] Seib C., Whiteside E., Voisey J., Lee K., Alexander K., Humphreys J., Chopin L., Anderson D. (2016). Stress, COMT polymorphisms, and depressive symptoms in older Australian women: An exploratory study. Genet. Test. Mol. Biomark..

[44] McQuaid R.J., McInnis O.A., Stead J.D., Matheson K., Anisman H. (2013). A paradoxical association of an oxytocin receptor gene polymorphism: Early-life adversity and vulnerability to depression. Front. Neurosci..

[45] Duncan L.E., Keller M.C. (2011). A critical review of the first 10 years of candidate gene-by-environment interaction research in psychiatry. Am. J. Psychiatry.

[46] Duncan L.E., Ostacher M., Ballon J. (2019). How genome-wide association studies (GWAS) made traditional candidate gene studies obsolete. Neuropsychopharmacology.

[47] Border R., Johnson E.C., Evans L.M., Smolen A., Berley N., Sullivan P.F., Keller M.C. (2019). No support for historic candidate gene or candidate gene-by-interaction hypotheses for major depression across multiple large samples. Am. J. Psychiatry.

[48] van de Weijer M.P., Pelt D.H.M., de Vries L.P., Baselmans B.M.L., Bartels M. (2022). A re-evaluation of candidate gene studies for well-being in light of genome-wide evidence. J. Happiness Stud..

[49] Wu S., Jia M., Ruan Y., Liu J., Guo Y., Shuang M., Gong X., Zhang Y., Yang X., Zhang D. (2005). Positive association of the oxytocin receptor gene (OXTR) with autism in the Chinese Han population. Biol. Psychiatry.

[50] Blomeyer D., Treutlein J., Esser G., Schmidt M.H., Schumann G., Laucht M. (2008). Interaction between CRHR1 gene and stressful life events predicts adolescent heavy alcohol use. Biol. Psychiatry.

[51] Miu A.C., Crisan L.G., Chis A., Ungureanu L., Druga B., Vulturar R. (2012). Somatic markers mediate the effect of serotonin transporter gene polymorphisms on Iowa Gambling Task. Genes Brain Behav..

[52] Wang C.K., Aleksic A., Xu M.S., Procyshyn R.M., Ross C.J., Vila-Rodriguez F., Ramos-Miguel A., Yan R., Honer W.G., Barr A.M. (2016). A tetra-primer amplification refractory system technique for the cost-effective and novel genotyping of eight single-nucleotide polymorphisms of the catechol-O-methyltransferase gene. Genet. Test. Mol. Biomark..

[53] Wang C.K., Xu M.S., Ross C.J., Lo R., Procyshyn R.M., Vila-Rodriguez F., White R.F., Honer W.G., Barr A.M. (2015). Development of a cost-efficient novel method for rapid, concurrent genotyping of five common single nucleotide polymorphisms of the brain derived neurotrophic factor (BDNF) gene by tetra-primer amplification refractory mutation system. Int. J. Methods Psychiatr. Res..

[54] Bernstein D.P., Fink L. (1998). Childhood Trauma Questionnaire: A retrospective Self-Report Manual.

[55] Taylor S.E., Lerner J.S., Sage R.M., Lehman B.J., Seeman T.E. (2004). Early environment, emotions, responses to stress, and health. J. Personal..

[56] Di Giuseppe R., Leaf R., Gorman B., Robin M.W. (2018). The development of a measure of irrational/rational beliefs. J. Ration.-Emotive Cogn.-Behav. Ther..

[57] Lindner H., Kirkby R., Wertheim E., Birch P. (1999). A brief assessment of irrational thinking: The shortened general attitude and belief scale. Cogn. Ther. Res..

[58] Caspi A., Hariri A.R., Holmes A., Uher R., Moffitt T.E. (2010). Genetic sensitivity to the environment: The case of the serotonin transporter gene and its implications for studying complex diseases and traits. Am. J. Psychiatry.

[59] Caspi A., McClay J., Moffitt T.E., Mill J., Martin J., Craig I.W., Taylor A., Poulton R. (2002). Role of genotype in the cycle of violence in maltreated children. Science.

[60] Caspi A., Sugden K., Moffitt T.E., Taylor A., Craig I.W., Harrington H., McClay J., Mill J., Martin J., Braithwaite A. (2003). Influence of life stress on depression: Moderation by a polymorphism in the 5-HTT gene. Science.

[61] Miu A.C., Homberg J.R., Lesch K.P. (2019). Genes, Brain, and Emotions: Interdisciplinary and Translational Perspectives.

[62] Rodriguez S., Gaunt T.R., Day I.N. (2009). Hardy-Weinberg equilibrium testing of biological ascertainment for Mendelian randomization studies. Am. J. Epidemiol..

[63] Keller M.C. (2014). Gene x environment interaction studies have not properly controlled for potential confounders: The problem and the (simple) solution. Biol. Psychiatry.

[64] Hayes A.F. (2022). Introduction to Mediation, Moderation, and Conditional Process Analysis: A Regression-Based Approach.

