# Supplementary material for: Gene–Environment Interactions in Irrational Beliefs: The Roles of Childhood Adversity and Multiple Candidate Genes
Source: Int J Mol Sci. 2024 Apr 10;25(8):4206. doi: 10.3390/ijms25084206 (PMC11050227; doi:10.3390/ijms25084206)

SERT_rs_25531

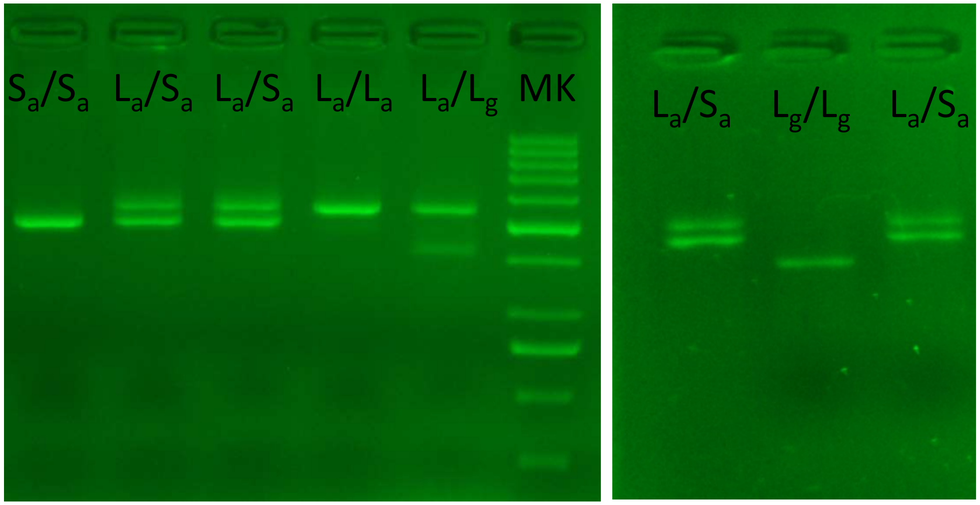

COMT_rs_6269

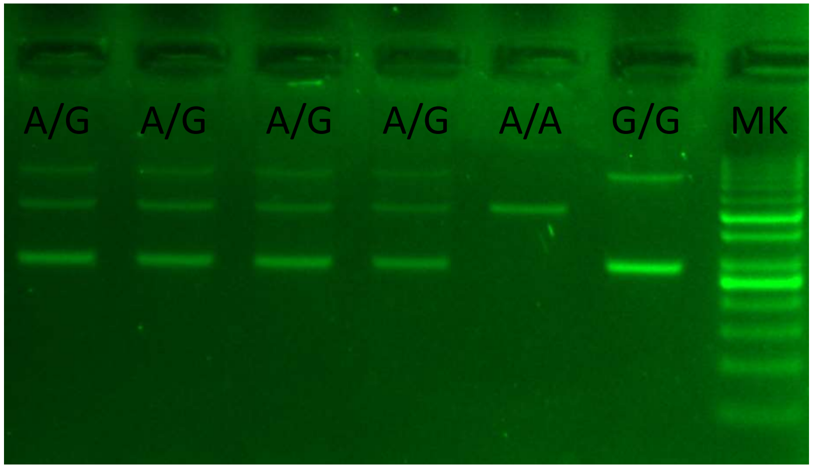

COMT_rs_737865

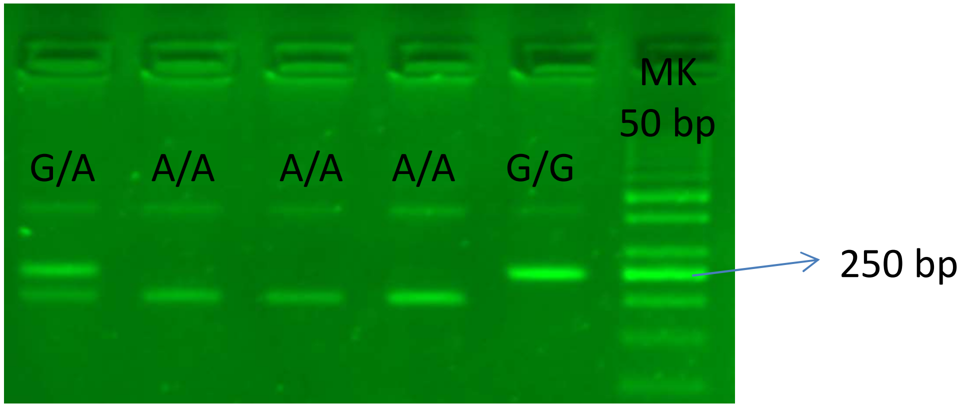

COMT_rs_165774

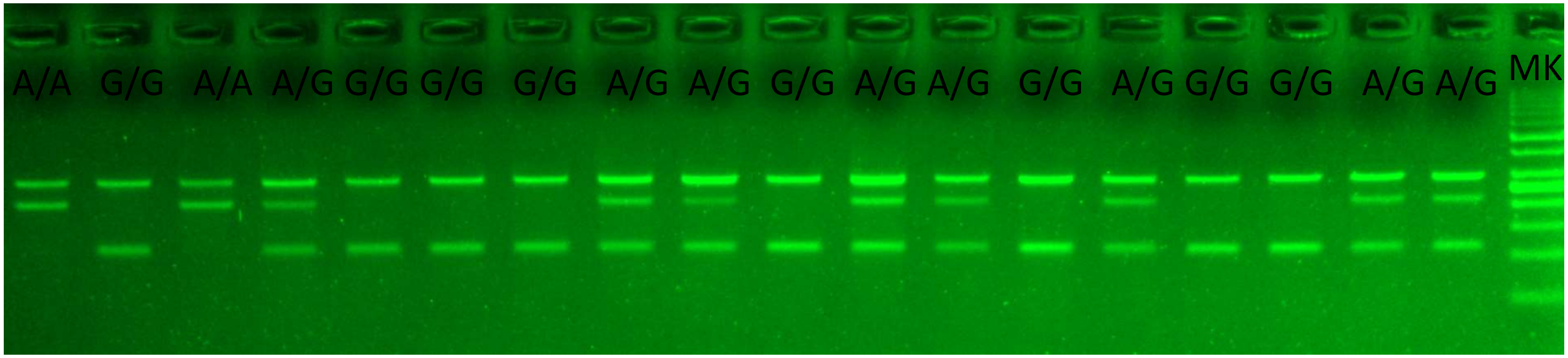

COMT_rs_2075507

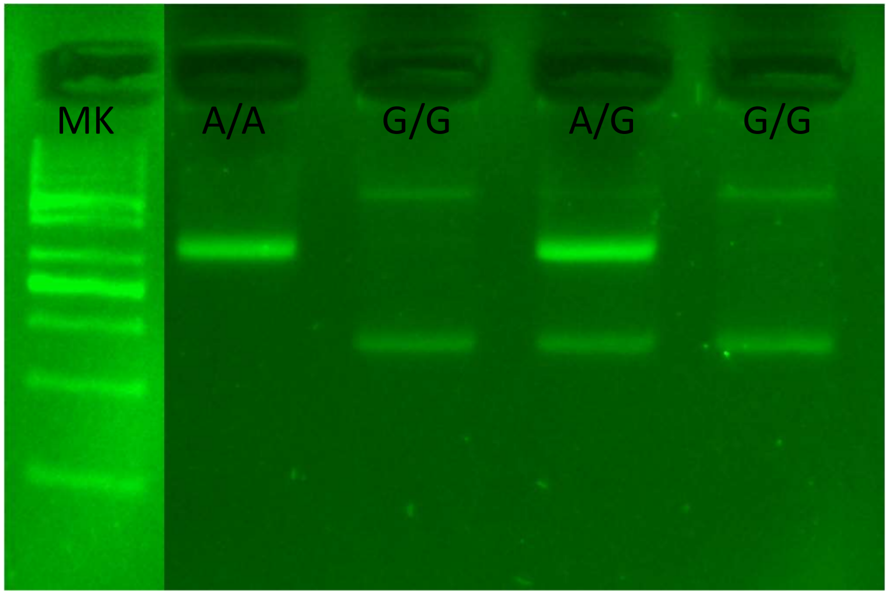

COMT_rs_4818

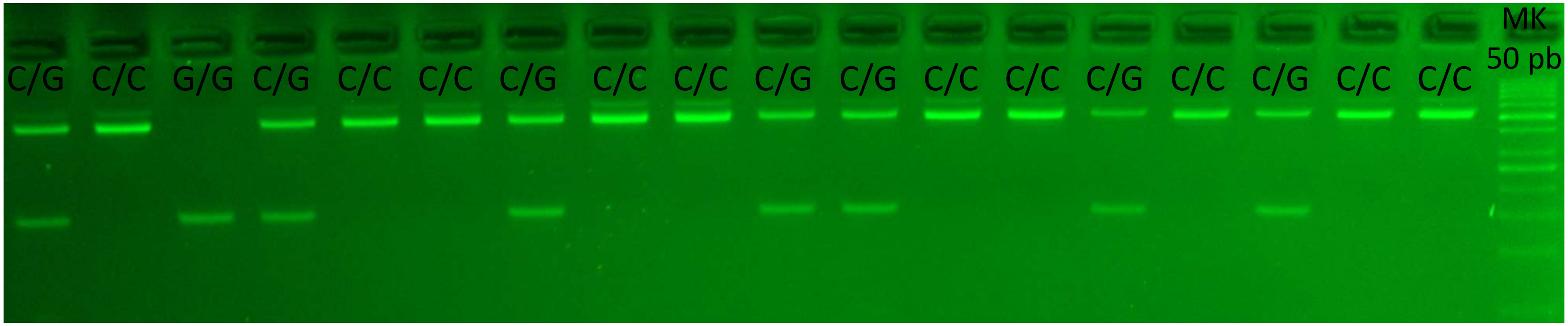

BDNF_rs_6265

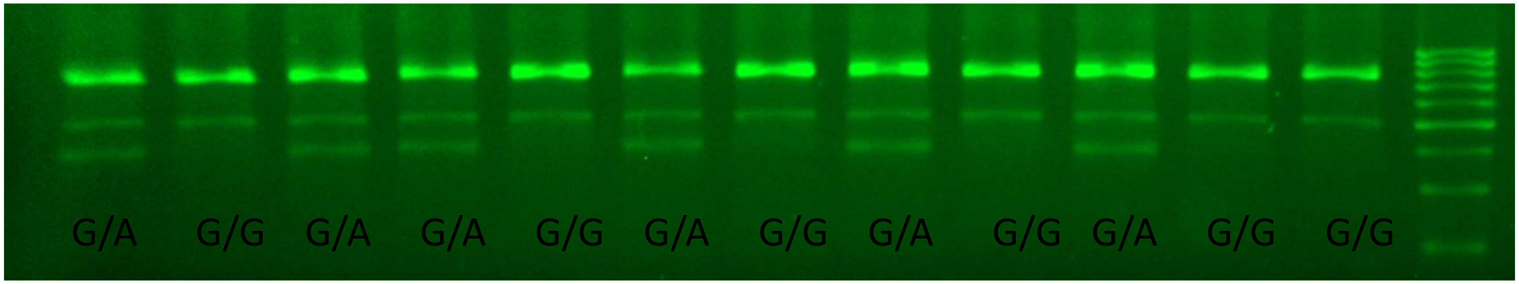

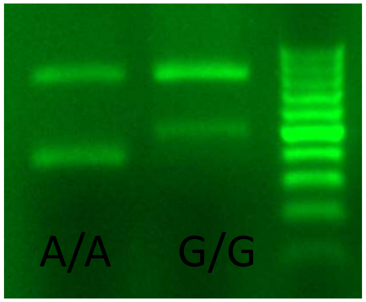

BDNF_rs_988748

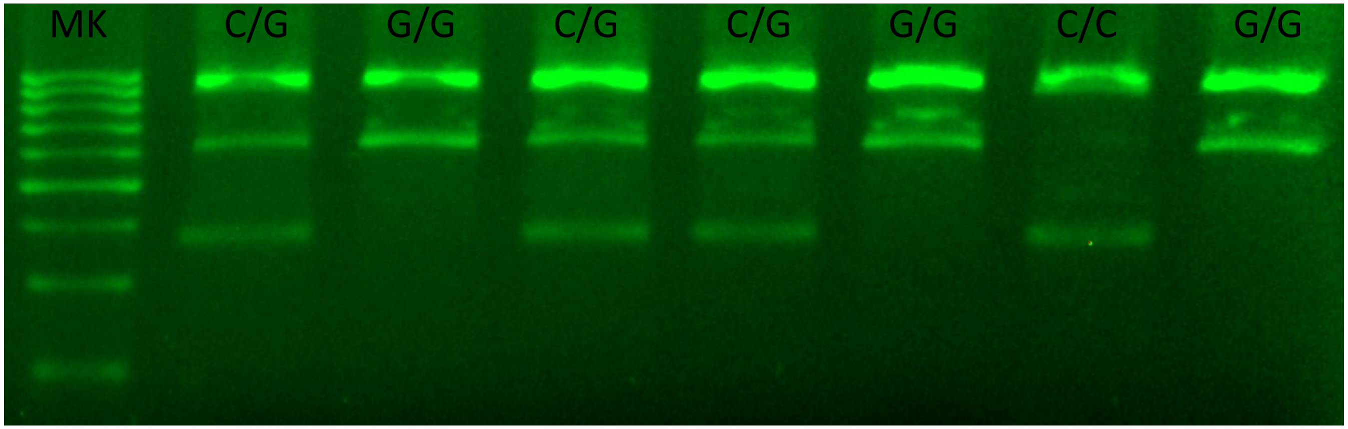

BDNF_rs_7103411

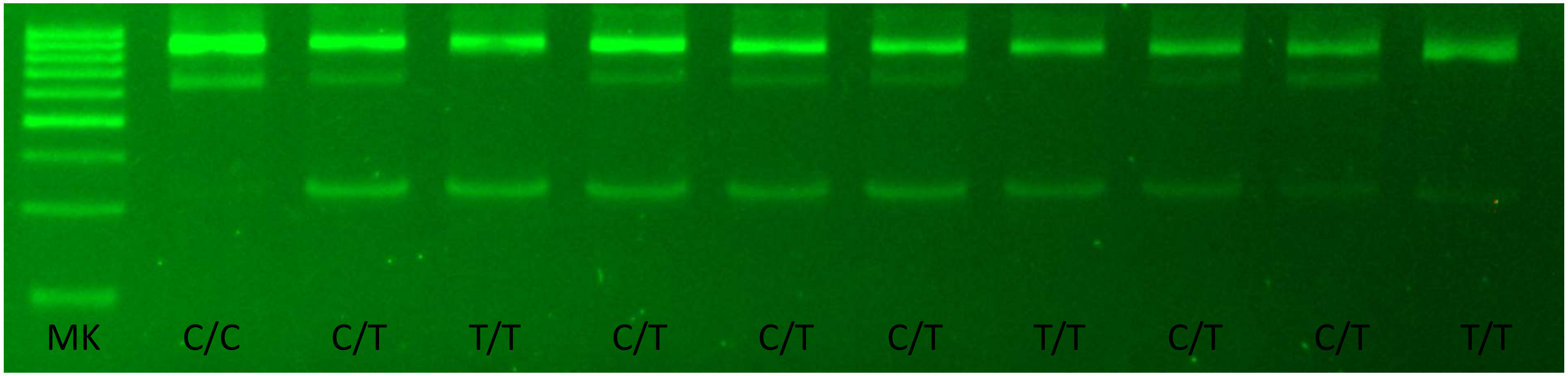

BDNF_rs_11030104

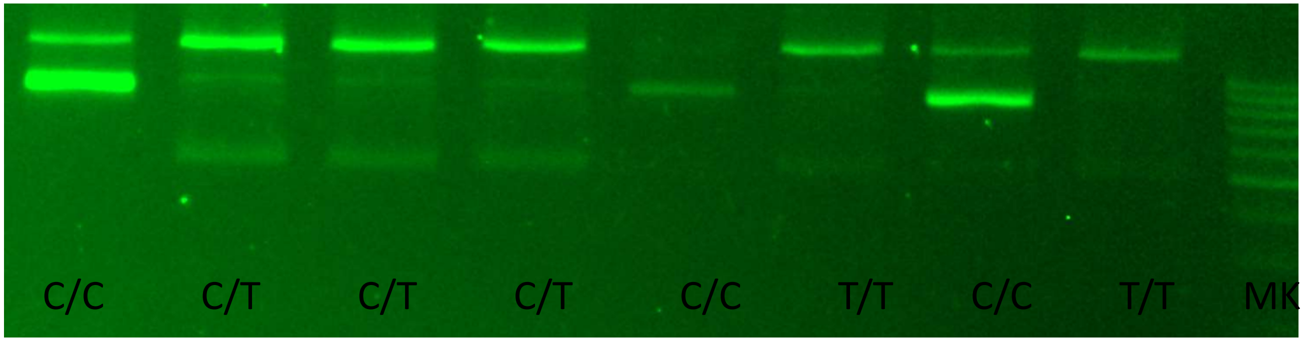

BDNF_rs_11757

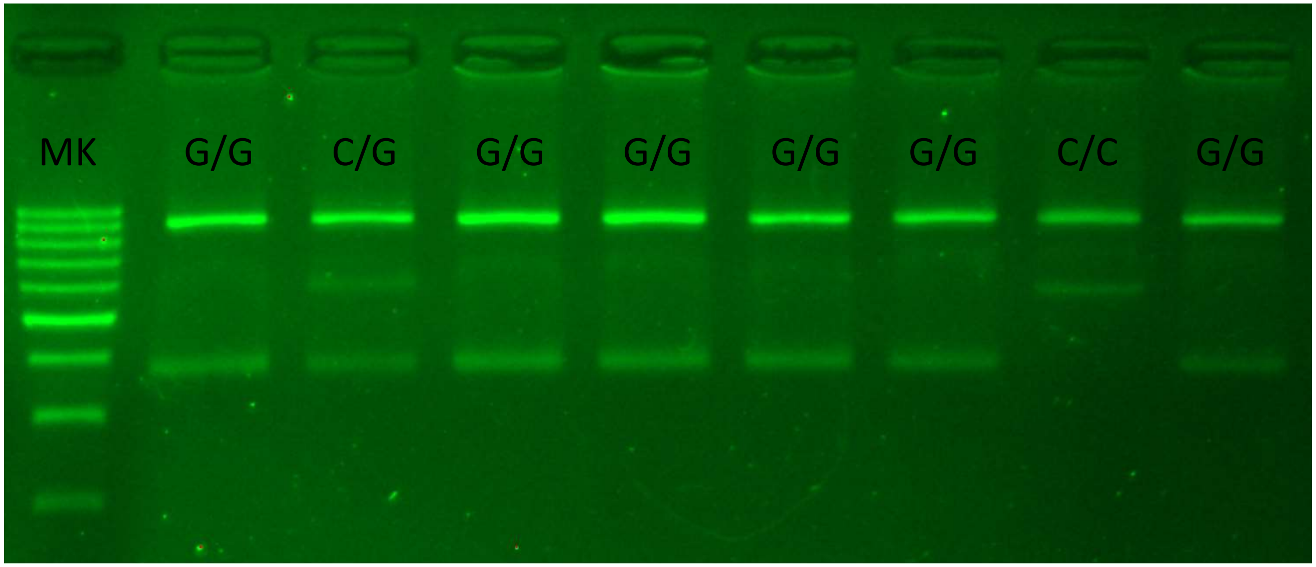

CRHR1_rs_242938

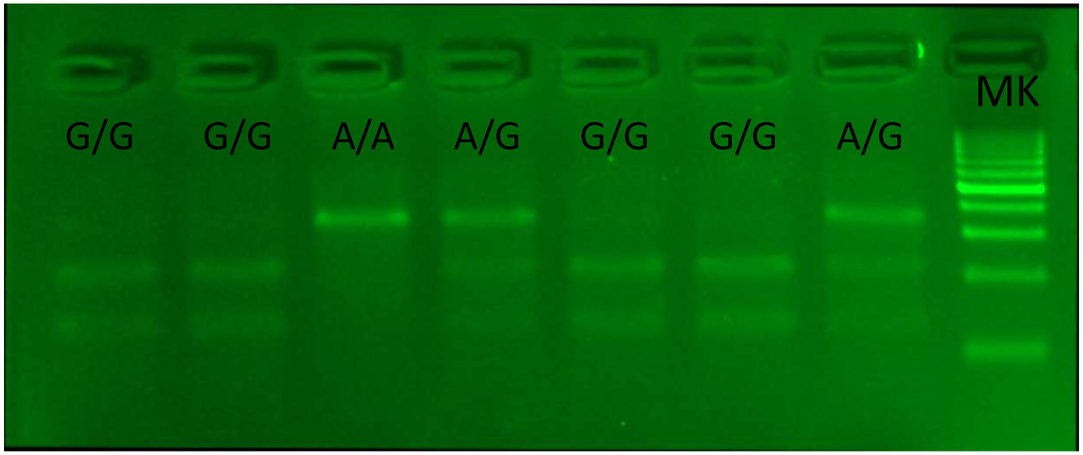

NR3C1_BclI_rs_41423247

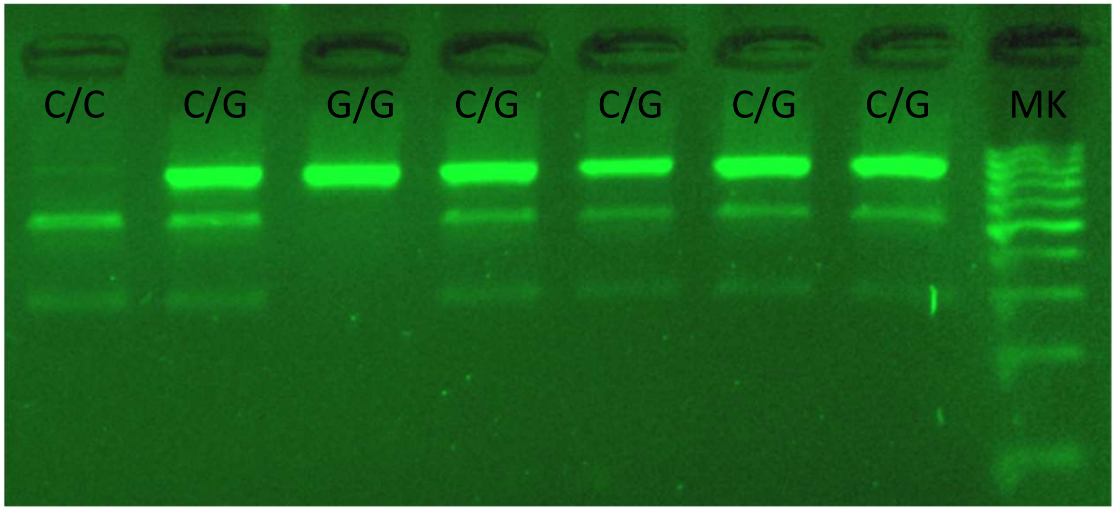

NR3C1_rs_6189_rs_6190

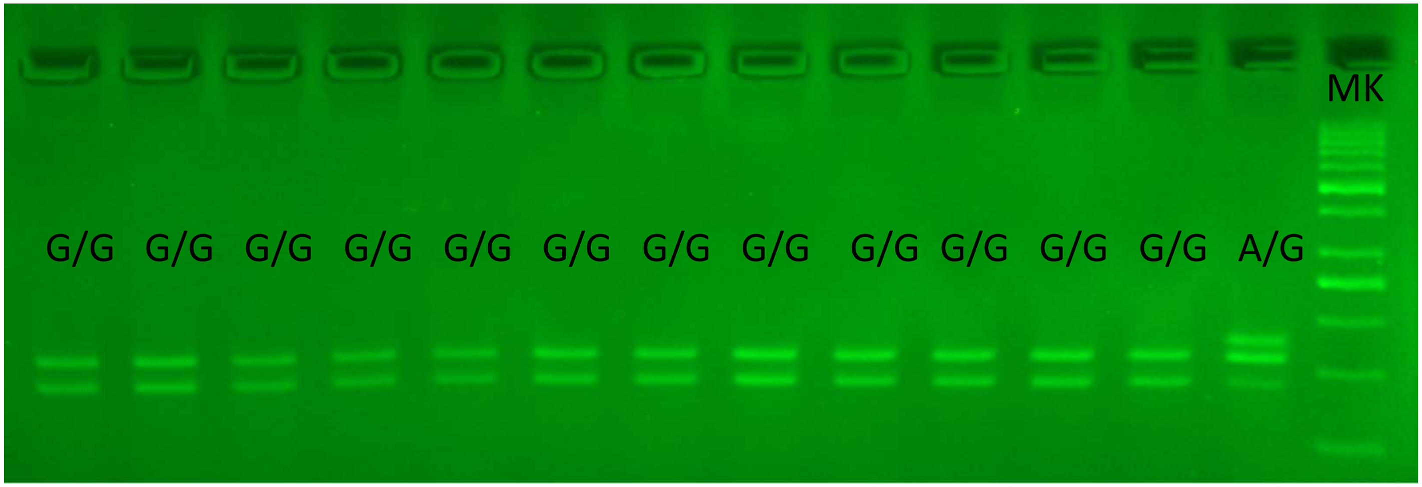

NR3C1_N363S_rs_56149945

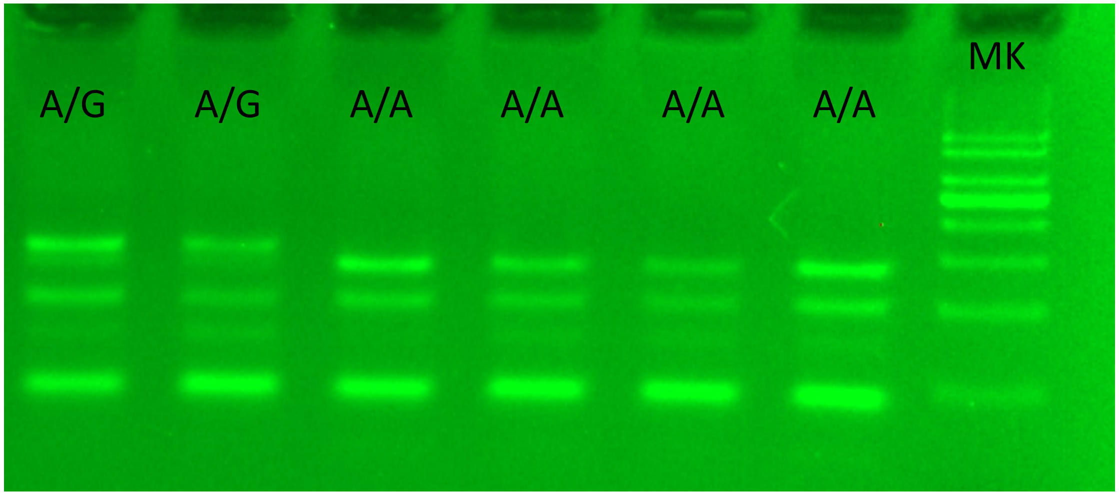

OXTR_rs_53576

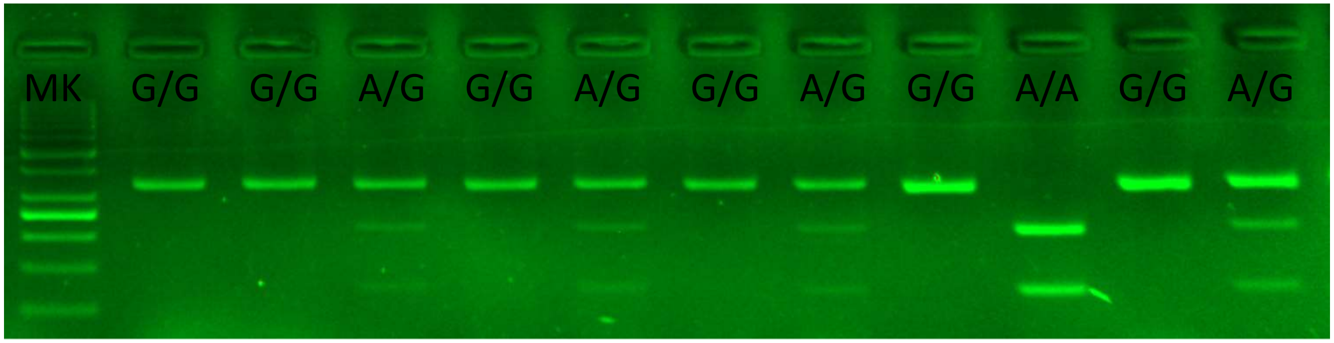

OXTR_rs_2254298

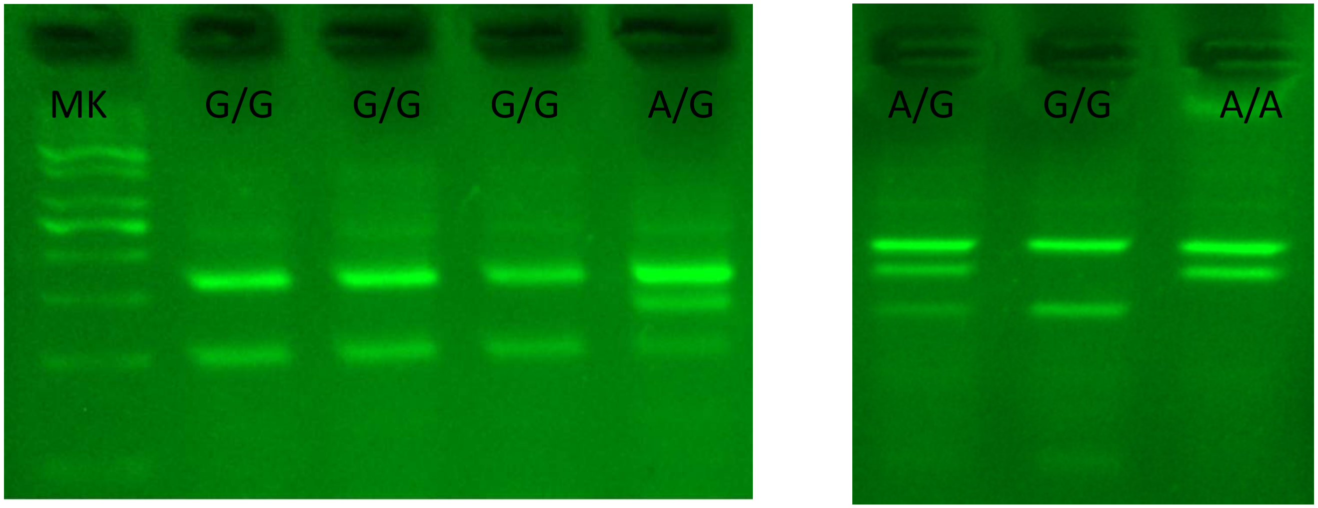

Supplement: Supplementary file 1 [file ijms-25-04206-s001.zip › Supplementary_Images.pdf]
