# Supplementary material for: Gene–Environment Interactions in Irrational Beliefs: The Roles of Childhood Adversity and Multiple Candidate Genes
Source: Int J Mol Sci. 2024 Apr 10;25(8):4206. doi: 10.3390/ijms25084206 (PMC11050227; doi:10.3390/ijms25084206)
Supplement: Supplementary file 1 [file ijms-25-04206-s001.zip › Supplementary_Tables SUBMIT R2.pdf]

Supplementary Table 1

Characteristics of the sample

Variable	
Sex distribution (% women)	84.73
Age	19.58 ± 3.53
CTQ-SF	38.82 ± 13.22
RFQ	1.82 ± 0.71
ABS-2 total irrationality	59.02 ± 25.71
GABS total irrationality	54.83 ± 15.78
PHQ-9	10.43 ± 5.84
GAD-7	8.65 ± 5.48

Note: Unless otherwise specified, all values indicate mean ± standard deviation.

Supplementary Table 2

The relations between childhood maltreatment and specific irrational beliefs

Childhood maltreatment	ABS-2							GABS					
	Demandingness	Self-downing	Low frustration tolerance	Awfulizing	Need for comfort	Need for achievement	Need for approval	Self-downing	Need for achievement	Need for approval	Need for comfort	Demands for fairness	Other-downing
CTQ-SF	.08 (N = 444)	.24** (N = 444)	.10* (N = 444)	.18** (N = 444)	.16** (N = 444)	.11* (N = 444)	.20** (N = 444)	.21** (N = 444)	.08 (N = 444)	.16** (N = 444)	.12** (N = 444)	.05 (N = 444)	.10** (N = 444)
RFQ	.04 (N = 450)	.18** (N = 450)	.09* (N = 452)	.13** (N = 450)	.15** (N = 452)	.08 (N = 450)	.12* (N = 450)	.18** (N = 445)	.08 (N = 447)	.12** (N = 445)	.09* (N = 445)	.04 (N = 445)	.09 (N = 445)

Note: CTQ-SF, Childhood Trauma Questionnaire – Short Form; ABS-2, Attitude and Belief Scale-2; GABS, General Attitudes and Belief Scale; RFQ, Risky Families Questionnaire. *, $p < 0.05$; ** $p < 0.01$.

Supplementary Table 3

Coefficients from models in which ABS-2 irrational beliefs was regressed on CTQ childhood maltreatment, genotypes, and their interactions

Model			B	SE (B)	P	95% CI
COMT	rs6269 (*N* = 304)	CTQ (centered)	0.346	0.163	0.035	0.025; 0.666
		Dummy 1 (AG vs. AA & GG)	3.347	3.101	0.281	-2.755; 9.449
		Dummy 2 (GG vs. AA & AG)	2.168	4.107	0. 598	-5.914; 10.250
		CTQ × Dummy 1	0.016	0.218	0.942	-0.412; 0.444
		CTQ × Dummy 2	-0.042	0.407	0.918	-0.844; 0.760
	rs737865 (*N* = 430)	CTQ (centered)	0.251	0.128	0.050	0.000; 0.502
		Dummy 1 (AG vs. AA & GG)	2.554	2.565	0.320	-2.488; 7.596
		Dummy 2 (GG vs. AA & AG)	1.350	4.213	0.749	-6.931; 9.632
		CTQ × Dummy 1	0.157	0.195	0.421	-0.226; 0.540
		CTQ × Dummy 2	0.106	0.322	0742	-0.526; 0.738
	rs165774 (*N* = 430)	CTQ (centered)	0.814	0.352	0.021	0.123; 1.506
		Dummy 1 (AG vs. AA & GG)	5.447	4.277	0.204	-2.961; 13.854
		Dummy 2 (GG vs. AA & AG)	0.095	4.268	0.982	-8.294; 8.484

		CTQ × Dummy 1	-0.342	0.371	0.358	-1.072; 0.388
		CTQ × Dummy 2	-0.861	0.383	0.025	-1.614; -0.108
	rs2075507 (*N* = 304)	CTQ (centered)	0.487	0.275	0.077	-0.054; 1.027
		Dummy 1 (AG vs. AA & GG)	0.946	3.835	0.805	-6.601; 8.493
		Dummy 2 (GG vs. AA & AG)	-2.188	3.936	0.579	-9.935; 5.558
		CTQ × Dummy 1	-0.266	0.313	0.396	-0.883; 0.350
		CTQ × Dummy 2	-0.021	0.320	0.947	-0.652; 0.609
	rs4818 (*N* = 304)	CTQ (centered)	0.224	0.145	0.123	-0.061; 0.510
		Dummy 1 (CG vs. CC & GG)	5.754	3.061	0.061	-0.271; 11.779
		Dummy 2 (GG vs. CC & CG)	0.284	4.035	0.944	-7.658; 8.225
		CTQ × Dummy 1	0.258	0.215	0.231	-0.165; 0.682
		CTQ × Dummy 2	0.256	0.390	0.513	-0.512; 1.023
NR3C1	N363S (*N* = 303)	CTQ (centered)	0.372	0.106	0.000	0.164; 0.580
		Dummy (AG vs. AA)	-1.517	6.004	0.801	-13.333; 10.300
		CTQ × Dummy	-0.320	0.527	0.544	-1.356; 0.717
	ER22/23EK (*N* = 304)	CTQ (centered)	0.357	0.111	0.001	0.140; 0.575
		Dummy (GA vs. GG)	-11.583	6.814	0.090	-24.991; 1.826
		CTQ × Dummy	0.173	0.318	0.587	-0.452; 0.798

OXTR	rs2254298 (*N* = 301)	CTQ (centered)	0.364	0.112	0.001	0.144; 0.584
		Dummy (AG vs. GG)	.250	3.726	0.946	-7.083; 7.584
		CTQ × Dummy	-0.036	0.284	0.898	-0.596; 0.523
	rs53576 (*N* = 430)	CTQ (centered)	0.468	0.139	0.001	0.196; 0.741
		Dummy 1 (AG vs. GG & AA)	1.498	2.572	0.561	-3.558; 6.553
		Dummy 2 (AA vs. GG & AG)	-1.570	4.051	0.699	-9.532; 6.392
		CTQ × Dummy 1	-0.238	0.194	0.220	-0.619; 0.143
		CTQ × Dummy 2	-0.327	0.311	0.294	-0.939; 0.285
CRHR1	rs242938 (*N* = 303)	CTQ (centered)	0.336	0.113	0.003	0.113; 0.559
		Dummy (GA vs. GG)	4.826	3.842	0.210	-2.735; 12.387
		CTQ × Dummy	0.092	0.278	0.742	-0.456; 0.639
SLC6A4	*5HTTLPR*/rs25531 (*N* = 304)	CTQ (centered)	0.511	0.232	0.028	0.055; 0.967
		Dummy 1 (L'S' vs. L'L' & S'S')	0.866	3.459	0.802	-5.941; 7.674
		Dummy 2 (S'S' vs. L'L' & L'S')	2.573	4.018	0.522	-5.335; 10.481
		CTQ × Dummy 1	-0.196	0.270	0.469	-0.728; 0.336
		CTQ × Dummy 2	-0.204	0.312	0.514	-0.819; 0.411
BDNF	rss6265 (*N* = 304)	CTQ (centered)	0.409	0.138	0.003	0.137; 0.681
		Dummy 1 (GA vs. GG & AA)	-0.233	2.986	0.938	-6.110; 5.643

		Dummy 2 (AA vs. GG & GA)	-2.363	6.243	0.705	-14.648; 9.923
		CTQ × Dummy 1	-0.307	0.221	0.166	-0.743; 0.129
		CTQ × Dummy 2	0.592	0.361	0.102	-0.119; 1.304
	rs988768 (*N* = 304)	CTQ (centered)	0.428	0.144	0.003	0.144; 0.711
		Dummy 1 (CG vs. GG & CC)	1.154	2.949	0.696	-4.649; 6.957
		Dummy 2 (CC vs. GG & CG)	-3.675	5.764	0.524	-15.018; 7.668
		CTQ × Dummy 1	-0.222	0.218	0.308	-0.650; 0.206
		CTQ × Dummy 2	0.227	0.380	0.551	-0.521; 0.975
	rs7103411 (*N* = 304)	CTQ (centered)	0.446	0.141	0.002	0.168; 0.725
		Dummy 1 (TC vs. TT & CC)	-0.286	2.915	0.922	-6.024; 5.451
		Dummy 2 (CC vs. TT & TC)	-5.410	7.611	0.478	-20.389; 9.569
		CTQ × Dummy 1	-0.235	0.212	0.269	-0.652; 0.183
		CTQ × Dummy 2	0.216	0.533	0.686	-0.833; 1.264
	rs1103014 (*N* = 304)	CTQ (centered)	0.429	0.144	0.003	0.146; 0.711
		Dummy 1 (TC vs. TT & CC)	0.879	2.968	0.767	-4.963; 6.720
		Dummy 2 (CC vs. TT & TC)	-1.471	5.297	0.781	-11.894; 8.952
		CTQ × Dummy 1	-0.309	0.220	0.161	-0.741; 0.124
		CTQ × Dummy 2	0.431	0.346	0.215	-0.251; 1.112

	rs11757 (*N* = 304)	CTQ (centered)	0.408	0.151	0.007	0.111; 0.705
		Dummy 1 (CG vs. GG & CC)	1.054	2.957	0.722	-4.766; 6.874
		Dummy 2 (CC vs. GG & CG)	-4.352	5.526	0.432	-15.226; 6.522
		CTQ × Dummy 1	-0.173	0.217	0.425	-0.599; 0.253
		CTQ × Dummy 2	0.263	0.378	0.487	-0.481; 1.008

Note: CTQ-SF, Childhood Trauma Questionnaire – Short Form; ABS-2, Attitude and Belief Scale-2.

Supplementary Table 4

Coefficients from models in which GABS irrational beliefs was regressed on CTQ childhood maltreatment, genotypes, and their interactions

Model			B	SE (B)	P	95% CI
COMT	rs6269 (*N* = 304)	CTQ (centered)	0.273	0.101	0.007	0.074; 0.471
		Dummy 1 (AG vs. AA & GG)	1.986	1.921	0.302	-1.793; 5.766
		Dummy 2 (GG vs. AA & AG)	3.972	2.544	0.119	-1.034; 8.978
		CTQ × Dummy 1	-0.078	0.135	0.561	-0.344; 0.187
		CTQ × Dummy 2	-0.153	0.252	0.545	-0.649; 0.344
	rs737865 (*N* = 430)	CTQ (centered)	0.167	0.079	0.035	0.012; 0.323
		Dummy 1 (AG vs. AA & GG)	1.716	1.590	0.281	-1.410; 4.841
		Dummy 2 (GG vs. AA & AG)	3.635	2.612	0.165	-1.498; 8.768
		CTQ × Dummy 1	0.065	0.121	0.588	-0.172; 0.303
		CTQ × Dummy 2	-0.062	0.199	0.757	-0.453; 0.330
	rs165774 (*N* = 430)	CTQ (centered)	0.570	0.218	0.009	0.142; 0.999
		Dummy 1 (AG vs. AA & GG)	2.931	2.651	0.270	-2.281; 8.142
		Dummy 2 (GG vs. AA & AG)	-0.071	2.646	0.979	-5.271; 5.129

		CTQ × Dummy 1	-0.286	0.230	0.214	-0.739; 0.166
		CTQ × Dummy 2	-0.631	0.237	0.008	-1.098; -0.165
	rs2075507 (*N* = 304)	CTQ (centered)	0.232	0.171	0.176	-0.104; 0.568
		Dummy 1 (AG vs. AA & GG)	0.630	2.386	0.792	-4.066; 5.326
		Dummy 2 (GG vs. AA & AG)	-1.912	2.449	0.436	-6.732; 2.908
		CTQ × Dummy 1	-0.047	0.195	0.809	-0.431; 0.337
		CTQ × Dummy 2	0.019	0.199	0.926	-0.374; 0.411
	rs4818 (*N* = 304)	CTQ (centered)	0.178	0.091	0.051	-0.001; 0.356
		Dummy 1 (CG vs. CC & GG)	2.892	1.912	0.131	-0.870; 6.655
		Dummy 2 (GG vs. CC & CG)	2.782	2.520	0.270	-2.177; 7.742
		CTQ × Dummy 1	0.095	0.134	0.479	-0.169; 0.360
		CTQ × Dummy 2	0.053	0.243	0.829	-0.426; 0.532
NR3C1	N363S (*N* = 303)	CTQ (centered)	0.232	0.066	0.000	0.103; 0.361
		Dummy (AG vs. AA)	3.038	3.727	0.416	-4.297; 10.373
		CTQ × Dummy	-0.321	0.327	0.327	-0.964; 0.323
	ER22/23EK (*N* = 304)	CTQ (centered)	0.214	0.069	0.002	0.079; 0.350
		Dummy (GA vs. GG)	-3.548	4.251	0.405	-11.914; 4.819
		CTQ × Dummy	0.093	0.198	0.641	-0.297; 0.483

OXTR	rs2254298 (*N* = 301)	CTQ (centered)	0.222	0.070	0.002	0.085; 0.360
		Dummy (AG vs. GG)	2.184	2.328	0.349	-2.399; 6.766
		CTQ × Dummy	-0.028	0.178	0.874	-0.378; 0.321
	rs53576 (*N* = 430)	CTQ (centered)	0.358	0.086	0.000	0.190; 0.526
		Dummy 1 (AG vs. GG & AA)	0.390	1.586	0.806	-2.728; 3.508
		Dummy 2 (AA vs. GG & AG)	-1.375	2.498	0.582	-6.286; 3.535
		CTQ × Dummy 1	-0.267	0.120	0.026	-0.502; -0.032
		CTQ × Dummy 2	-0.439	0.192	0.023	-0.816; -0.062
CRHR1	rs242938 (*N* = 303)	CTQ (centered)	0.213	0.070	0.003	0.075; 0.352
		Dummy (GA vs. GG)	3.114	2.388	0.193	-1.584; 7.813
		CTQ × Dummy	0.005	0.173	0.978	-0.335; 0.345
SLC6A4	*5HTTLPR*/rs25531 (*N* = 304)	CTQ (centered)	0.285	0.144	0.049	0.001; 0.568
		Dummy 1 (L'S' vs. L'L' & S'S')	0.335	2.152	0.876	-3.900; 4.570
		Dummy 2 (S'S' vs. L'L' & L'S')	1.264	2.500	0.614	-3.656; 6.183
		CTQ × Dummy 1	-0.069	0.168	0.683	-0.400; 0.262
		CTQ × Dummy 2	-0.119	0.194	0.542	-0.501; 0.264
BDNF	rss6265 (*N* = 304)	CTQ (centered)	0.215	0.086	0.013	0.045; 0.385
		Dummy 1 (GA vs. GG & AA)	1.005	1.865	0.590	-2.665; 4.674

		Dummy 2 (AA vs. GG & GA)	0.020	3.898	0.996	-7.651; 7.691
		CTQ × Dummy 1	-0.080	0.138	0.562	-0.352; 0.192
		CTQ × Dummy 2	0.336	0.226	0.138	-0.108; 0.780
	rs988768 (*N* = 304)	CTQ (centered)	0.234	0.090	0.010	0.057; 0.410
		Dummy 1 (CG vs. GG & CC)	0.632	1.838	0.731	-2.986; 4.250
		Dummy 2 (CC vs. GG & CG)	-0.522	3.594	0.885	-7.594; 6.550
		CTQ × Dummy 1	-0.061	0.136	0.653	-0.328; 0.206
		CTQ × Dummy 2	0.121	0.237	0.609	-0.345; 0.588
	rs7103411 (*N* = 304)	CTQ (centered)	0.252	0.088	0.005	0.078; 0.425
		Dummy 1 (TC vs. TT & CC)	-0.062	1.816	0.973	-3.637; 3.513
		Dummy 2 (CC vs. TT & TC)	-3.170	4.742	0.504	-12.502; 6.163
		CTQ × Dummy 1	-0.082	0.132	0.535	-0.342; 0.178
		CTQ × Dummy 2	0.043	0.332	0.896	-0.610; 0.696
	rs1103014 (*N* = 304)	CTQ (centered)	0.237	0.089	0.009	0.061; 0.413
		Dummy 1 (TC vs. TT & CC)	1.002	1.850	0.588	-2.638; 4.642
		Dummy 2 (CC vs. TT & TC)	0.050	3.301	0.988	-6.445; 6.546
		CTQ × Dummy 1	-0.123	0.137	0.369	-0.393; 0.146
		CTQ × Dummy 2	0.275	0.216	0.204	-0.150; 0.699

	rs11757 (*N* = 304)	CTQ (centered)	0.201	0.094	0.034	0.015; 0.386
		Dummy 1 (CG vs. GG & CC)	0.745	1.844	0.687	-2.884; 4.374
		Dummy 2 (CC vs. GG & CG)	-0.645	3.445	0.852	-7.425; 6.135
		CTQ × Dummy 1	0.007	0.135	0.961	-0.259; 0.272
		CTQ × Dummy 2	0.161	0.236	0.496	-0.303; 0.625

Note: CTQ-SF, Childhood Trauma Questionnaire – Short Form; GABS, General Attitudes and Belief Scale.

Supplementary Table 5

Coefficients from models in which ABS-2 irrational beliefs was regressed on RFQ childhood maltreatment, genotypes, and their interactions

Model			B	SE (B)	P	95% CI
COMT	rs6269 (*N* = 309)	RFQ (centered)	3.204	3.286	0.330	-3.263; 9.670
		Dummy 1 (AG vs. AA & GG)	3.080	3.104	0.322	-3.028; 9.188
		Dummy 2 (GG vs. AA & AG)	1.909	4.149	0.646	-6.256; 10.074
		RFQ × Dummy 1	3.185	4.312	0.461	-5.301; 11.671
		RFQ × Dummy 2	1.945	7.805	0.803	-13.414; 17.303
	rs737865 (*N* = 438)	RFQ (centered)	3.925	2.462	0.112	-0.913; 8.763
		Dummy 1 (AG vs. AA & GG)	2.509	2.574	0.330	-2.550; 7.568
		Dummy 2 (GG vs. AA & AG)	0.918	4.185	0.826	-7.308; 9.145
		RFQ × Dummy 1	1.556	3.630	0.668	-5.579; 8.691
		RFQ × Dummy 2	5.491	6.010	0.361	-6.321; 17.303
	rs165774 (*N* = 438)	RFQ (centered)	12.142	5.845	0.038	0.655; 23.630
		Dummy 1 (AG vs. AA & GG)	5.497	4.324	0.204	-3.002; 13.995
		Dummy 2 (GG vs. AA & AG)	-0.579	4.311	0.893	-9.052; 7.894

		RFQ × Dummy 1	-2.995	6.327	0.636	-15.431; 9.441
		RFQ × Dummy 2	-13.063	6.389	0.042	-25.621; -0.505
	rs2075507 (*N* = 309)	RFQ (centered)	5.340	4.477	0.234	-3.470; 14.150
		Dummy 1 (AG vs. AA & GG)	1.055	3.863	0.785	-6.548; 8.657
		Dummy 2 (GG vs. AA & AG)	-1.863	3.958	0.638	-9.653; 5.926
		RFQ × Dummy 1	-1.598	5.409	0.768	-12.241; 9.046
		RFQ × Dummy 2	1.417	5.639	0.802	-9.679; 12.513
	rs4818 (*N* = 309)	RFQ (centered)	1.813	3.083	0.557	-4.254; 7.881
		Dummy 1 (CG vs. CC & GG)	5.125	3.070	0.096	-0.916; 11.166
		Dummy 2 (GG vs. CC & CG)	0.192	4.073	0.962	-7.822; 8.207
		RFQ × Dummy 1	5.112	4.229	0.228	-3.209; 13.434
		RFQ × Dummy 2	7.768	7.628	0.309	-7.242; 22.779
NR3C1	N363S (*N* = 308)	RFQ (centered)	5.620	2.097	0.008	1.493; 9.746
		Dummy (AG vs. AA)	-1.177	5.976	0.844	-12.937; 10.583
		RFQ × Dummy	-6.139	8.264	0.458	-22.401; 10.124
	ER22/23EK (*N* = 309)	RFQ (centered)	4.644	2.121	0.029	0.471; 8.817
		Dummy (GA vs. GG)	-10.871	6.720	0.107	-24.095; 2.353
		RFQ × Dummy	8.463	6.977	0.226	-5.266; 22.192

OXTR	rs2254298 (*N* = 306)	RFQ (centered)	4.592	2.179	0.036	0.304; 8.880
		Dummy (AG vs. GG)	0.727	3.754	0.847	-6.659; 8.113
		RFQ × Dummy	2.684	5.793	0.643	-8.716; 14.084
	rs53576 (*N* = 438)	RFQ (centered)	7.584	2.517	0.003	2.636; 12.531
		Dummy 1 (AG vs. GG & AA)	2.029	2.571	0.430	-3.024; 7.082
		Dummy 2 (AA vs. GG & AG)	-0.260	4.124	0.950	-8.365; 7.845
		RFQ × Dummy 1	-2.539	3.655	0.488	-9.722; 4.644
		RFQ × Dummy 2	-11.926	5.614	0.034	-22.960; -0.891
CRHR1	rs242938 (*N* = 308)	RFQ (centered)	4.335	2.159	0.046	0.086; 8.584
		Dummy (GA vs. GG)	5.092	3.855	0.188	-2.494; 12.677
		RFQ × Dummy	6.079	6.056	0.316	-5.837; 17.996
SLC6A4	*5HTTLPR*/rs25531 (*N* = 309)	RFQ (centered)	7.600	3.887	0.051	-0.050; 15.250
		Dummy 1 (L'S' vs. L'L' & S'S')	1.452	3.447	0.674	-5.331; 8.235
		Dummy 2 (S'S' vs. L'L' & L'S')	3.570	4.022	0.376	-4.346; 11.485
		RFQ × Dummy 1	-3.026	4.804	0.529	-12.479; 6.427
		RFQ × Dummy 2	-4.395	5.869	0.454	-15.943; 7.153
BDNF	rss6265 (*N* = 309)	RFQ (centered)	6.402	2.611	0.015	1.264; 11.539
		Dummy 1 (GA vs. GG & AA)	0.384	2.969	0.897	-5.460; 6.227

		Dummy 2 (AA vs. GG & GA)	-0.437	6.186	0.944	-12.609; 11.735
		RFQ × Dummy 1	-6.513	4.248	0.126	-14.872; 1.846
		RFQ × Dummy 2	19.748	8.909	0.027	2.216; 37.280
	rs988768 (*N* = 309)	RFQ (centered)	6.872	2.691	0.011	1.576; 12.167
		Dummy 1 (CG vs. GG & CC)	1.654	2.953	0.576	-4.157; 7.466
		Dummy 2 (CC vs. GG & CG)	-1.180	5.687	0.836	-12.371; 10.010
		RFQ × Dummy 1	-5.379	4.301	0.212	-13.843; 3.086
		RFQ × Dummy 2	3.648	7.978	0.648	-12.052; 19.348
	rs7103411 (*N* = 309)	RFQ (centered)	7.187	2.618	0.006	2.036; 12.338
		Dummy 1 (TC vs. TT & CC)	0.122	2.918	0.967	-5.620; 5.865
		Dummy 2 (CC vs. TT & TC)	-5.740	7.647	0.453	-20.788; 9.308
		RFQ × Dummy 1	-5.403	4.250	0.205	-13.766; 2.960
		RFQ × Dummy 2	-1.484	10.752	0.890	-22.642; 19.673
	rs1103014 (*N* = 309)	RFQ (centered)	6.730	2.721	0.014	1.376; 12.085
		Dummy 1 (TC vs. TT & CC)	1.437	2.977	0.630	-4.421; 7.296
		Dummy 2 (CC vs. TT & TC)	1.254	5.251	0.811	-9.079; 11.587
		RFQ × Dummy 1	-6.032	4.303	0.162	-14.499; 2.435
		RFQ × Dummy 2	7.716	7.443	0.301	-6.931; 22.362

	rs11757 (*N* = 309)	RFQ (centered)	6.263	2.868	0.030	0.619; 11.907
		Dummy 1 (CG vs. GG & CC)	1.600	2.969	0.590	-4.243; 7.444
		Dummy 2 (CC vs. GG & CG)	-2.172	5.492	0.693	-12.978; 8.634
		RFQ × Dummy 1	-3.612	4.239	0.395	-11.953; 4.729
		RFQ × Dummy 2	4.350	7.917	0.583	-11.228; 19.929

Note: ABS-2, Attitude and Belief Scale-2; RFQ, Risky Families Questionnaire.

Supplementary Table 6

Coefficients from models in which GABS irrational beliefs was regressed on RFQ childhood maltreatment, genotypes, and their interactions

Model			B	SE (B)	P	95% CI
COMT	rs6269 (N = 304)	RFQ (centered)	3.039	2.044	0.138	-0.984; 7.062
		Dummy 1 (AG vs. AA & GG)	2.081	1.933	0.283	-1.723; 5.885
		Dummy 2 (GG vs. AA & AG)	4.338	2.599	0.096	-0.776; 9.452
		RFQ × Dummy 1	1.019	2.685	0.705	-4.265; 6.303
		RFQ × Dummy 2	0.235	4.865	0.961	-9.338; 9.809
	rs737865 (N = 431)	RFQ (centered)	2.220	1.537	0.150	-0.802; 5.242
		Dummy 1 (AG vs. AA & GG)	1.835	1.594	0.250	-1.298; 4.968
		Dummy 2 (GG vs. AA & AG)	3.584	2.625	0.173	-1.576; 8.743
		RFQ × Dummy 1	0.753	2.284	0.742	-3.736; 5.241
		RFQ × Dummy 2	1.795	3.738	0.631	-5.552; 9.142
	rs165774 (N = 431)	RFQ (centered)	6.844	3.601	0.058	-0.234; 13.921
		Dummy 1 (AG vs. AA & GG)	3.162	2.671	0.237	-2.088; 8.412
		Dummy 2 (GG vs. AA & AG)	-0.056	2.664	0.983	-5.293; 5.180

		RFQ × Dummy 1	-1.440	3.909	0.713	-9.122; 6.243
		RFQ × Dummy 2	-8.144	3.954	0.040	-15.915; -0.372
	rs2075507 (*N* = 304)	RFQ (centered)	3.121	2.776	0.262	-2.341; 8.584
		Dummy 1 (AG vs. AA & GG)	0.902	2.400	0.707	-3.822; 5.627
		Dummy 2 (GG vs. AA & AG)	-1.734	2.464	0.482	-6.584; 3.116
		RFQ × Dummy 1	-0.261	3.364	0.938	-6.881; 6.360
		RFQ × Dummy 2	1.371	3.506	0.696	-5.529; 8.271
	rs4818 (*N* = 304)	RFQ (centered)	1.984	1.929	0.305	-1.812; 5.780
		Dummy 1 (CG vs. CC & GG)	2.785	1.923	0.148	-0.998; 6.569
		Dummy 2 (GG vs. CC & CG)	3.172	2.566	0.217	-1.877; 8.221
		RFQ × Dummy 1	2.485	2.650	0.349	-2.730; 7.699
		RFQ × Dummy 2	4.379	4.783	0.361	-5.033; 13.792
NR3C1	N363S (*N* = 303)	RFQ (centered)	3.719	1.312	0.005	1.137; 6.300
		Dummy (AG vs. AA)	2.867	3.710	0.440	-4.435; 10.168
		RFQ × Dummy	-2.860	5.132	0.578	-12.959; 7.238
	ER22/23EK (*N* = 304)	RFQ (centered)	3.137	1.332	0.019	0.517; 5.758
		Dummy (GA vs. GG)	-3.292	4.184	0.432	-11.527; 4.942
		RFQ × Dummy	4.663	4.348	0.284	-3.894; 13.220

OXTR	rs2254298 (*N* = 301)	RFQ (centered)	3.324	1.371	0.016	0.626; 6.022
		Dummy (AG vs. GG)	2.346	2.345	0.318	-2.269; 6.961
		RFQ × Dummy	0.704	3.618	0.846	-6.415; 7.823
	rs53576 (*N* = 431)	RFQ (centered)	5.004	1.611	0.002	1.839; 8.170
		Dummy 1 (AG vs. GG & AA)	0.430	1.592	0.787	-2.698; 3.559
		Dummy 2 (AA vs. GG & AG)	-0.867	2.532	0.732	-5.844; 4.110
		RFQ × Dummy 1	-2.487	2.291	0.278	-6.991; 2.016
		RFQ × Dummy 2	-9.540	3.475	0.006	-16.371; -2.709
CRHR1	rs242938 (*N* = 303)	RFQ (centered)	3.386	1.353	0.013	0.723; 6.049
		Dummy (GA vs. GG)	3.214	2.399	0.181	-1.507; 7.934
		RFQ × Dummy	0.712	3.767	0.850	-6.701; 8.126
SLC6A4	*5HTTLPR*/rs25531 (*N* = 304)	RFQ (centered)	3.946	2.421	0.104	-0.819; 8.711
		Dummy 1 (L'S' vs. L'L' & S'S')	0.516	2.161	0.812	-3.737; 4.769
		Dummy 2 (S'S' vs. L'L' & L'S')	1.584	2.508	0.528	-3.353; 6.520
		RFQ × Dummy 1	-0.108	3.004	0.971	-6.020; 5.803
		RFQ × Dummy 2	-1.892	3.651	0.605	-9.078; 5.293
BDNF	rss6265 (*N* = 304)	RFQ (centered)	3.621	1.647	0.029	0.380; 6.862
		Dummy 1 (GA vs. GG & AA)	0.946	1.874	0.614	-2.742; 4.635

		Dummy 2 (AA vs. GG & GA)	0.950	3.869	0.806	-6.664; 8.565
		RFQ × Dummy 1	-1.888	2.677	0.481	-7.155; 3.380
		RFQ × Dummy 2	9.627	5.575	0.085	-1.345; 20.599
	rs988768 (*N* = 304)	RFQ (centered)	3.967	1.689	0.020	0.642; 7.291
		Dummy 1 (CG vs. GG & CC)	0.630	1.848	0.733	-3.007; 4.268
		Dummy 2 (CC vs. GG & CG)	-0.033	3.611	0.993	-7.139; 7.074
		RFQ × Dummy 1	-1.934	2.688	0.472	-7.224; 3.356
		RFQ × Dummy 2	3.715	5.061	0.463	-6.245; 13.675
	rs7103411 (*N* = 304)	RFQ (centered)	4.411	1.646	0.008	1.171; 7.652
		Dummy 1 (TC vs. TT & CC)	-0.035	1.827	0.985	-3.631; 3.561
		Dummy 2 (CC vs. TT & TC)	-3.377	4.759	0.479	-12.743; 5.989
		RFQ × Dummy 1	-2.417	2.657	0.364	-7.646; 2.811
		RFQ × Dummy 2	-0.512	6.690	0.939	-13.678; 12.654
	rs1103014 (*N* = 304)	RFQ (centered)	3.987	1.705	0.020	0.632; 7.343
		Dummy 1 (TC vs. TT & CC)	1.025	1.860	0.582	-2.634; 4.685
		Dummy 2 (CC vs. TT & TC)	0.842	3.316	0.800	-5.684; 7.368
		RFQ × Dummy 1	-2.782	2.684	0.301	-8.064; 2.500
		RFQ × Dummy 2	6.622	4.698	0.160	-2.624; 15.868

	rs11757 ($N = 304$)	RFQ (centered)	3.345	1.803	0.064	-0.203; 6.893
		Dummy 1 (CG vs. GG & CC)	0.719	1.859	0.699	-2.939; 4.378
		Dummy 2 (CC vs. GG & CG)	-0.264	3.476	0.940	-7.104; 6.576
		RFQ × Dummy 1	-0.418	2.652	0.875	-5.636; 4.801
		RFQ × Dummy 2	4.098	5.010	0.414	-5.761; 13.958

Note: GABS, General Attitudes and Belief Scale; RFQ, Risky Families Questionnaire.

Supplementary Table 7

Allelic frequencies in the European population, according to the dbSNP (https://www.ncbi.nlm.nih.gov/snp/)

Gene polymorphism	Allele (frequency %) in Europeans		Sample size (no. of genotyped participants)	Reference
COMT rs6269	A (60.19)	G (39.8)	32946	https://www.ncbi.nlm.nih.gov/snp/rs6269
COMT rs737865	A (70.5)	G (29.45)	140924	https://www.ncbi.nlm.nih.gov/snp/rs737865
COMT rs165774	G (68.62)	A (31.37)	98852	https://www.ncbi.nlm.nih.gov/snp/rs165774
COMT rs2075507	G (44.75)	A (55.25)	13768	https://www.ncbi.nlm.nih.gov/snp/rs2075507
COMT rs4818	C (60.89)	G (39.10)	36934	https://www.ncbi.nlm.nih.gov/snp/rs4818
NR3C1 BclI	G (63.04)	C (36.95)	14286	https://www.ncbi.nlm.nih.gov/snp/rs41423247
NR3C1 N363S	T (96.79	C (3.2)	214000	https://www.ncbi.nlm.nih.gov/snp/rs56149945
NR3C1 ER22/23EK	C (97.52)	G (2.47)	285418	https://www.ncbi.nlm.nih.gov/snp/rs6190
OXTR rs2254298	G (88.71)	A (11.28)	32396	https://www.ncbi.nlm.nih.gov/snp/rs2254298
OXTR rs53576	A (32.57)	G (67.42)	62252	https://www.ncbi.nlm.nih.gov/snp/rs53576
CRHR1 rs242938	A (7.51)	G (92.48)	14286	https://www.ncbi.nlm.nih.gov/snp/rs242938
SLC6A4 5HTTLPR/rs25531	T or L_A (97.87)	C or L_G (2.13%)	8684	https://www.ncbi.nlm.nih.gov/snp/rs25531
BDNF rs6265	C (80.62)	T (19.37)	336012	https://www.ncbi.nlm.nih.gov/snp/rs6265
BDNF rs988748	C (20.39)	G (79.60)	14420	https://www.ncbi.nlm.nih.gov/snp/rs988748
BDNF rs7103411	C (22.51)	T (77.48)	127900	https://www.ncbi.nlm.nih.gov/snp/rs7103411
BDNF rs1103014	A (37.39)	G (62.60)	27100	https://www.ncbi.nlm.nih.gov/snp/rs622628
BDNF rs11757	C (72.76)	G (27.23)	17594	https://www.ncbi.nlm.nih.gov/snp/rs11757
